# The prevalence and proportion of asymptomatic tuberculosis among adolescents and young adults: a systematic review and meta-analysis

**DOI:** 10.1186/s12879-026-14156-y

**Published:** 2026-08-04

**Authors:** Mohammad E. Heidari, Nicolas Ngo, Chawangwa Modongo, Amyn A. Malik, Maria R. Jaswal, Adrienne E. Shapiro, Rebecca H. Berhanu, Neil A. Martinson, Limakatso Lebina, Stefan Niemann, James A. Seddon, Sanghyuk S. Shin

**Affiliations:** 1 Sue & Bill Gross School of Nursing, University of California Irvine, Irvine, CA USA; 2 Victus Global Botswana Organization, Gaborone, Botswana; 3 O’Donnell School of Public Health, UT Southwestern Medical Center, Dallas, TX USA; 4 IRD Global, Singapore, Singapore; 5 Baitussalam Welfare Trust, Karachi, Pakistan; 6 Department of Global Health, University of Washington, Seattle, WA USA; 7 Division of Allergy and Infectious Diseases, Department of Medicine, University of Washington, Seattle, WA USA; 8 University of the Witwatersrand, Johannesburg, South Africa; 9 Department of Medicine, Division of Infectious Diseases, Vanderbilt University Medical Center, Nashville, TN USA; 10 Perinatal HIV Research Unit, University of the Witwatersrand, Johannesburg, USA; 11 Johns Hopkins University Center for TB Research, Baltimore, USA; 12 Africa Health Research Institute, Clinical Trials Unit, KwaZulu-Natal, South Africa; 13 Research Center Borstel, Leibniz Lung Center, Borstel, Germany; 14 German Center for Infection Research, Partner Site Hamburg-Lübeck-Borstel-Riems, Borstel, Germany; 15 EPHE, Université des Antilles, Paris, France; 16 Desmond Tutu TB Centre, Department of Pediatrics and Child Health, Stellenbosch University, Cape Town, South Africa; 17 Department of Infectious Disease, Imperial College London, London, UK

**Keywords:** Asymptomatic, Tuberculosis, Adolescents, Young adults, Subclinical

## Abstract

**Background:**

Recent studies report ≥ 50% of people with tuberculosis (TB) have asymptomatic disease, which is likely to be infectious. Asymptomatic TB among adolescents and young adults (AYAs; defined as individuals aged 10 to 24 years) has been poorly studied. To estimate the prevalence of asymptomatic TB in the population of adolescents and young adults and the proportion of TB that remains asymptomatic in this group.

**Methods:**

A systematic review and meta-analysis were conducted using PubMed, Web of Science, Scopus, and CINAHL from inception to October 2025. Studies reporting asymptomatic TB among AYAs were included. Study quality was assessed using the Joanna Briggs Institute (JBI) Critical Appraisal Checklist. Outcomes included (1) prevalence (asymptomatic TB cases among those screened) and (2) proportion (asymptomatic TB cases among diagnosed TB cases). Pooled estimates were calculated using mixed-effects meta-analysis. The review was registered in PROSPERO (CRD420250652821).

**Results:**

Of 3,185 articles initially found, sixteen studies were included after full-text review. Included studies were conducted in Africa (*n* = 8), Asia (*n* = 7) and Europe (*n* = 1). The pooled prevalence of asymptomatic TB from 11 included studies among AYAs was 1% (95% confidence interval [CI]: 1%-3%), driven by high prevalence among household contact studies. The pooled proportion of asymptomatic TB from 12 included studies among AYAs with TB was 62% (95% CI: 40% -80%).

**Conclusion:**

We found high prevalence and proportion of asymptomatic TB among AYAs in many regions, underlining the importance of symptom-agnostic screening and preventative strategies in high-risk populations.

**Supplementary Information:**

The online version contains supplementary material available at https://doi.org/10.1186/s12879-026-14156-y.

## Introduction

Tuberculosis (TB) remains a substantial global public health challenge, with an estimated 10.7 million people falling ill with TB globally and 1.23 million dying from the disease in 2024. Approximately 60% of individuals developing TB globally in 2023 were adolescents, young adults, and adults (aged 15–39) [1]. Adolescents and young adults (AYAs; defined as those aged 10–24 years) are at high risk for TB, treatment complications, and adverse outcomes [2]. Despite this, TB in AYAs has long been a neglected field of research and public health action [3, 4]. TB in this age group may be driven by environmental as well as societal factors, including conditions that are prone to overcrowding, such as school, dormitories, and community gathering centers, are major risk factors for exposure as well as facilitating transmission of *Mycobacterium tuberculosis* among AYAs [5, 6]. Notwithstanding recent concerns for AYAs with TB, there are important knowledge gaps related to asymptomatic infection in AYAs, which may be a hidden pool fueling community transmission.

Asymptomatic TB occurs in people where *Mycobacterium tuberculosis* can be isolated from their sputum despite the individual being asymptomatic. This form of TB poses several challenges since it has the potential to cause undetected transmission until it reaches a stage where it becomes clinically apparent [7–10]. Traditional passive case-finding approaches, which rely on individuals presenting to healthcare facilities with symptoms, are insufficient to detect these subclinical or asymptomatic cases. Active case-finding (ACF) in community settings without relying on symptom screening remains the only effective approach to identify those with asymptomatic TB [11–13]. There is an urgent need to better understand the epidemiology of asymptomatic TB, particularly among AYAs, to guide focused ACF strategies for this population. This study, therefore, aims to review the literature on the prevalence of asymptomatic TB among AYAs.

## Methods

### Search strategy and selection criteria

This systematic review was conducted according to the Preferred Reporting Items for Systematic Reviews and Meta-Analyses (PRISMA) 2020 guidelines [14] and registered on PROSPERO (registration ID: CRD420250652821). A comprehensive search strategy was performed by searching electronic databases: PubMed, Web of Science, Scopus, and Cumulative Index to Nursing and Allied Health Literature (CINAHL) from inception to October 2025 with focus on the English language or papers with an English abstract. We also targeted grey literature sources including Open Access Theses and Dissertations (OTAD), medRxiv, ProQuest, World Health Organization (WHO), and Centers for Disease Control and Prevention (CDC) databases. Search terms included combinations of keywords and MeSH terms related to “subclinical tuberculosis,” “asymptomatic tuberculosis,” “TB,” “Youth,” “adolescent,” “college student, “child” and “prevalence.” Because most of the existing papers included children and adolescents as one category, we included the search terms related to children as well as adolescents. The full search strategy is listed in Supplementary Table S1. We searched for bibliographies of all relevant studies and attempted to contact authors if a study appeared potentially eligible but did not report the data required.

Two reviewers (M.H, N.N) screened titles and abstracts of all potentially eligible publications in Covidence software with support from a third reviewer (S.S), using the following criteria: (1) Studies that considered individuals aged between 10 and 24 years, including adolescents [8–17], and young adults [18–22]; and (2) studies that reported diagnoses of asymptomatic TB. For studies that enrolled AYAs as part of a broader age range but did not provide age-disaggregated results, we contacted the authors to request AYA-specific numerators and denominators. If authors did not respond or were unable to provide this data, the study was excluded from quantitative analysis. Asymptomatic TB was defined as individuals with a microbiologic diagnosis of TB disease in which *Mycobacterium tuberculosis* was detectable in sputum, but the participant did not report TB symptoms. We restricted TB diagnoses to microbiologically confirmed pulmonary TB, defined as ≥ 1 positive sputum test by smear microscopy, nucleic-acid amplification test (NAAT), or culture (liquid or solid). Individuals diagnosed on clinical or radiographic grounds alone, or via non-sputum tests such as urine lipoarabinomannan, were not categorized as confirmed TB. We also considered studies that focused on case finding and the screening of individuals. We excluded studies with screening algorithms that rely solely on symptom screening, as such studies would not be able to provide unbiased estimates of asymptomatic TB proportions. A few studies included only asymptomatic TB at baseline which were included for prevalence estimation. Exclusion criteria included qualitative studies, editorial papers, and papers for which the full text paper was not accessible. We also excluded papers conducted solely in clinical and hospital settings, which focused on clinical diagnosis, even if they reported subclinical or asymptomatic TB. Longitudinal studies which excluded people with TB at baseline, but included people who developed TB during follow up, were not considered for estimating prevalence, but were included for proportion evaluation. After exclusion of studies according to these criteria, full text articles were assessed for inclusion by the same reviewers.

### Data extraction and assessment

A standardized data extraction form was developed in a Microsoft Excel spreadsheet. Two reviewers (M.H., N.N.) extracted and cross-checked data independently for each included study. The quality of included studies was assessed by using the Joanna Briggs Institute (JBI) Critical Appraisal Checklist for observational studies (Supplementary Table S2). Studies were rated as low risk if ≥ 7 items were assessed as “Yes” and all critical items were satisfied; moderate risk if 5–6 items were “Yes” or if one critical item was “Unclear”; and high risk if < 5 items were “Yes” or if any critical item was rated “No.”

### Statistical analysis

We calculated two primary outcomes: (1) the prevalence of asymptomatic TB, defined as the number of individuals with bacteriologically confirmed TB but no clinical symptoms divided by the total number screened, and (2) the proportion of asymptomatic TB among all individuals with TB identified. Prevalence estimates with 95% confidence intervals (CIs) were pooled using a GLMM-based random-effects meta-analysis, and between-study heterogeneity was quantified using Q, I², and τ² statistics estimated by maximum likelihood [15]. Subgroup meta-analysis and meta-regressions were also performed to try to identify potential sources of between-study variation. Leave-one-out sensitivity analysis was used to examine influence on aggregated estimates and on between-studies variation. Publication bias was measured using visual evaluation of funnel plots, Egger’s regression test, Begg’s test for rank correlation, and a trim and fill analysis. All calculations were done in R software version 4.5.1 using packages meta and metafor [16, 17].

**Fig. 1:**
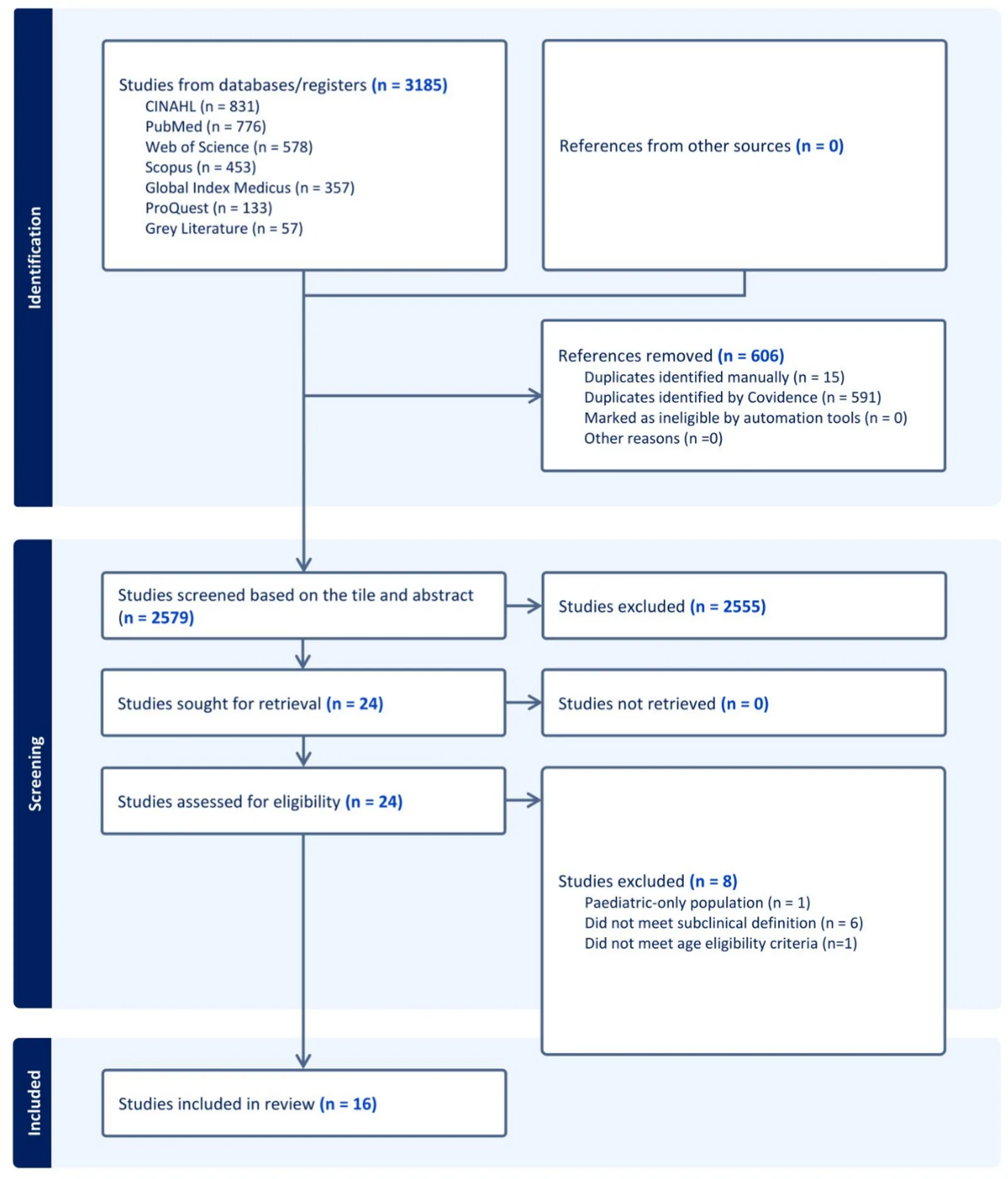
PRISMA flow chart to evaluate the prevalence of asymptomatic TB in adolescents and young adults (AYAs)

## Results

A total of 3,185 records were identified through database searches and grey literature sources. After removing duplicates, 2,579 records remained for title and abstract screening. Following the screening process, 24 full-text articles were assessed for eligibility, of which 16 published between 2011 and 2025 covering regions in Africa (*n* = 8), Asia (*n* = 7) and Europe (*n* = 1) met inclusion criteria and were included in this systematic review (Fig. 1). The mean age of participants was 19.32 (3.82) years old, with some studies focused on adolescents (10–19 years), some on young adults (20–24 years) and some both.

### Settings and screening

Study designs of the 16 included studies including school-based screenings, household contact investigations, community prevalence surveys, and facility-based cohorts (Table 1). Six studies implemented ACF with universal bacteriological testing for all screened participants regardless of symptoms or chest X-ray (CXR). Eleven studies incorporated CXR in their ACF process. In contrast, ten studies used a conditional bacteriological approach testing only if they had abnormal CXR or clinical symptoms [18, 19, 23–30] and the information for one study was not available [31]. Artificial intelligence (AI) assisted CXR was reported in four studies [24–26, 29]. Household contact screening was the focus of eight included studies [20–23, 26, 30, 32, 33]. Within this household subset, five studies used universal bacteriological testing.

School settings were less commonly studied but provide unique insights into adolescent TB transmission. Two included studies were conducted in a school setting. Dorjee et al. [18] evaluated the Zero TB Kids initiative in Tibetan refugee boarding schools in India, implementing annual systematic screening with tuberculin skin tests, CXR, and molecular diagnostics. Another school-based investigation by Qian Wu [19] in China targeted junior and high schools after TB outbreaks, identifying 12 students aged 15 years with TB, 11 of whom were asymptomatic. HIV status of participants was reported in nine studies [18, 20–22, 26, 27, 33– 30]. These comprised community, clinic, and household-based investigations in South Africa, and Asia.

### Risk of bias assessment

Using the JBI Critical Appraisal Checklist, 11 studies were judged to have low risk of bias, 3 were identified as moderate risk, and 2 had high risk, based on the domains (Supplementary Table S3). Prevalence of asymptomatic TB among 9 studies with low risk of bias was 1% (95% CI: 0%-2%; I² = 97.1%, τ² = 1.54) (Supplementary Figure S1). Proportion of asymptomatic TB among 8 studies with low risk of bias was 73% (95% CI: 44%-90%; I² = 86.2%, τ² = 2.45) (Supplementary Figure S2). The pooled prevalence and proportion of TB based on moderate and high risk of bias are shown in Supplementary Figure S3-S5.

**Table 1:** Study characteristics of the included articles

Authors	Year	Country	Total Population Screened/ AYAs Population Screened (10–24 y)	AYAs age category	Setting	Included CXR in the screening	Universal microbiologic sputum test	Symptom/CXR triage with microbiology for screen-positives only	Computer aided detection using CXR
Mendelsohn SC et al.	2025	South Africa	979/232	18–24 years old	Household-based	No	Yes	No	No
Odume B et al.	2024	Nigeria	8,516/1,277	15–24 years old	Community-based	Yes	No	Yes	Yes
Innes AL et al.	2024	Viet Nam	96,631/NA	10–24 years old	Household-based	Yes	No	Yes	Yes
Ananda NR et al.	2023	Indonesia	321/8	10–18 years old	Community-based	Yes	No	Yes	Yes
Wang Z et al.	2023	China	506/39	18–24 years	Prison-based	Yes	No	Yes	No
Berhanu RH et al.	2023	South Africa	30,513/3412	18–24 years old	Facility-based, Household-based	No	Yes	No	No
Jaswal MR et al.	2023	Pakistan	21,035/14,564	10–24 years old	Household-based	Yes	No	Yes	No
Carter N et al.	2023	South Africa	2077/799	10–24 years old	Household-based	No	Yes	No	No
Wu Q et al.	2023	China	1213	10–18 years old	School-based	Yes	No	Yes	No
Govender I et al.	2022	South Africa	12,375/5292	15–24 years old	Household-based, Community-based, Clinic-based	Yes	No	Yes	Yes
Mendelsohn SC et al.	2022	South Africa	861/83	18–24 years old	Household-based	No	Yes	No	No
Dorjee K et al.	2021	India	6,582	10–19 years old	School-based	Yes	No	Yes	No
Gunasekera K et al.	2020	South Africa	30,375/5,966	18–24 years old	Community-based	Yes	Yes	NA	No
Nair A et al.	2016	India	151/NA	20–24 years old	Clinic-based	Yes	No	Yes	No
Shapiro AE et al.	2012	South Africa	1540	10–24 years old	Household-based, Community- based	No	Yes	No	No
Castela Torres da Costa J et al.	2011	Portugal	6,112/NA	20–24 years old	Clinic-based	Yes	No	Yes	No

TB: Tuberculosis, AYAs: Adolescent and young adults, CXR: Chest X-ray, ACF: Active case finding, Xpert MTB/RIF: Rapid molecular test for TB diagnosis, TST: Tuberculin Skin Test, HIV: human immunodeficiency viruses, PTB: Pulmonary tuberculosis, NAAT: Nucleic Acid Amplification Test, IGRA: Interferon-Gamma Release Assay, AI: Artificial intelligence, NA: Not available

### Meta analysis of prevalence of asymptomatic TB

**Fig. 2:**
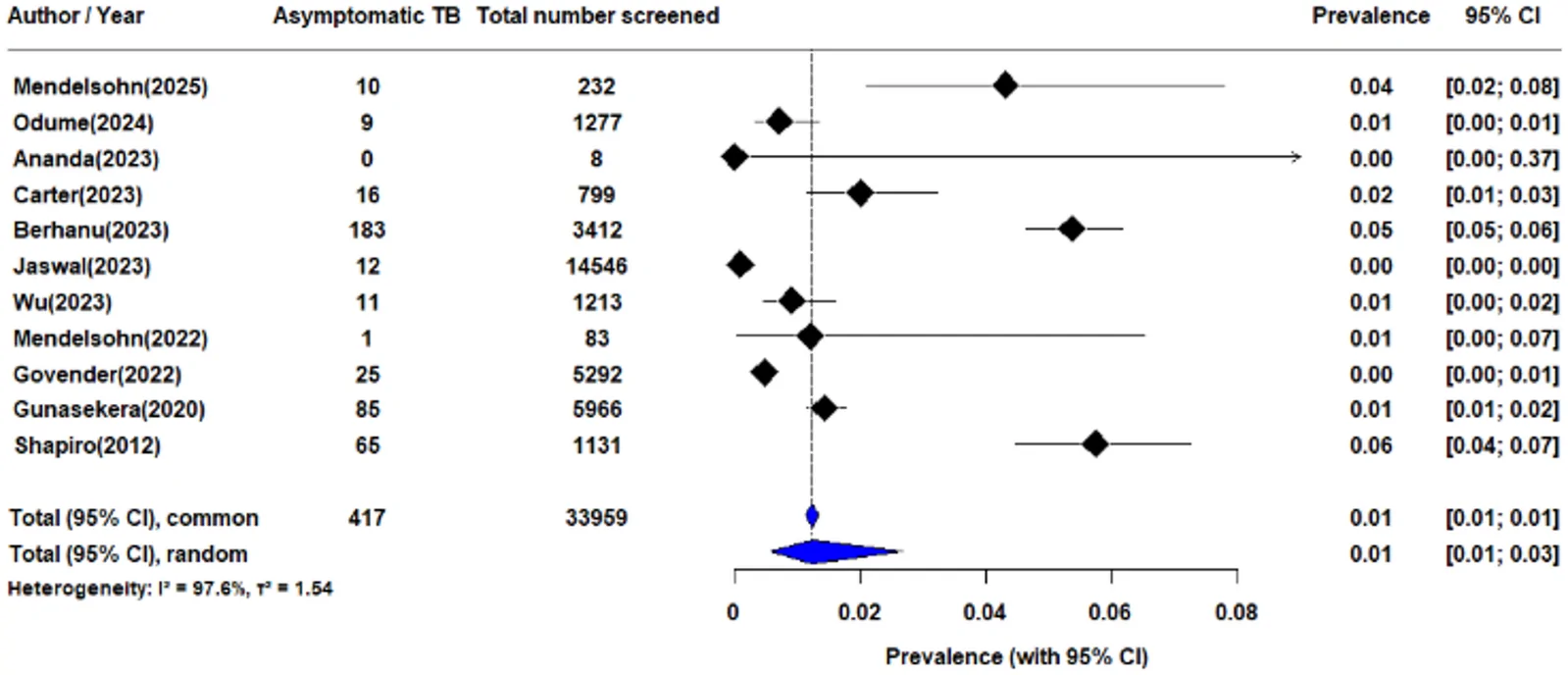
Pooled prevalence of asymptomatic tuberculosis among adolescents and young adults (AYAs)

Eleven studies were included for estimating prevalence with 33,959 participants and 417 individuals with TB. The pooled prevalence of asymptomatic TB among AYAs under the random effect model was 1% (95% CI: 1%-3%; *I² = 97.6%*,* τ² = 1.54*) (Fig. 2). We performed the subgroup analysis among AYAs based on the WHO age category, among studies which were conducted in the last 5 years and by geographical region. The pooled prevalence among adolescents (10–19 years old) was 1% and among young people (20–25 years old) was 2% (Supplementary Figures S6 and S7). The pooled prevalence of asymptomatic TB was 2% (95% CI: 1%-5%) among studies published in the last 5 years (Supplementary Figure S8). The prevalence of asymptomatic TB among AYAs in African and Asian countries were 2% and 0.19%, respectively (Supplementary Figures S9 and S10).

### Meta-analysis of proportion of asymptomatic TB

**Fig. 3:**
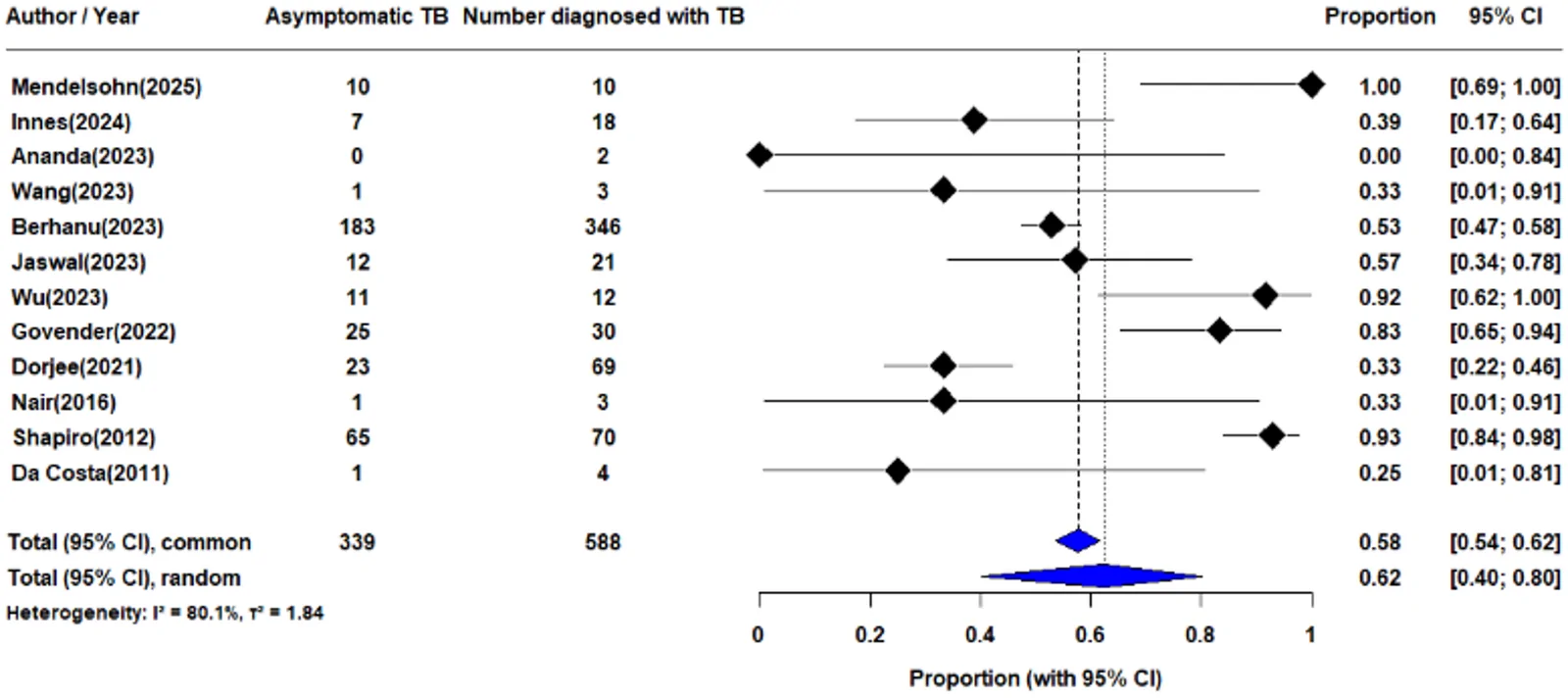
Pooled proportion of asymptomatic tuberculosis among adolescents and young adults (AYAs) with TB

A total of 12 studies (588 participants, 339 events) were included. The pooled proportion of asymptomatic TB among AYAs with TB was 62% (95% CI: 40%-80%). Between-study heterogeneity was substantial (*I² = 80.1%*,* τ² = 1.84*), and Q test was significant (*p* < 0.0001) (Fig. 3). The pooled proportion of asymptomatic TB among AYAs with TB in the last 5 years was 63% (95% CI: 27%-89%; *I² = 24%*,* τ² = 2.7*) (Supplementary Figure S11). Among adolescents, the pooled proportion was 48% (*I²=75.8%*,* τ² = 3.12*) (Supplementary Figure S12) and among young adults it was 52% (95% CI: 47%-57%; *I²=0*,* τ² =0*) (Supplementary Figure S13). The proportion of asymptomatic TB among AYAs in African and Asian countries were 86% (95% CI: 61%-96%; *I²=91.1%*,* τ²=1.38*) and 47% (95% CI: 28%-66%; *I²=45.5*,* τ² =0.61*), respectively (Supplementary Figures S14 and S15).

### Estimation based on the setting and ACF algorithm

A random-effects meta-analysis of six studies (*n* = 11,623) estimated a pooled prevalence of asymptomatic TB at 3% (95% CI 2%–5%; *I² = 96.1%*,* τ² = 0.33*) among studies which applied the universal bacteriological test (Supplementary Figure S16). Of note, five of the six studies that used universal bacteriological testing are household contact studies. The pooled prevalence of asymptomatic TB was 0.3% (95% CI 0.13%-0.72%; *I² = 88.7%*,* τ² = 0.83*) among studies in which bacteriological testing was conditional on screening positive with CXR and/or symptoms (Supplementary Figure S17). For the proportion outcome, a random-effects meta-analysis of three studies applying universal bacteriological test estimated an overall pooled proportion of 88% (95% CI: 48%–98%), with between-study heterogeneity *(I² = 92.4%*,* τ² = 2.28*, *p* *< 0.001*) (supplementary Figure S18). Nine studies adopted conditional bacteriological tests, and the overall pooled proportion was at 51% (95% CI: 31%–71%; *I² = 69%*,* τ² = 0.99*) (supplementary Figure S19).

Seven household contact studies (*n* = 25,495) estimated a pooled prevalence of 1% (95% CI 0%–4%; *I² = 98.2%*,* τ² = 2.17*) (supplementary Figure S20). The pooled proportion of asymptomatic TB among household studies (*n* = 495) was 76% (95% CI 51%-90%; *I² = 86.2%*,* τ² = 1.47*, *p* *< 0.001*) (supplementary Figure S21).

### Meta-regression

In multivariable meta-regression analysis including mean age and region, neither variable was significantly associated with asymptomatic TB prevalence among adolescents and young adults (AYA), although studies from Asia reporting lower prevalence than those from Africa (β = − 1.40, *I² = 94.36%*) (Supplementary Tables S4-S6). In the multivariable meta-regression model, the proportion of asymptomatic TB among AYA was associated with mean age (β = −0.38, CI: -0.53, -0.24, *I² = 0%*), but not region (β = −0.24, CI: -2.47, 1.98) (Supplementary Tables S6-9S).

### Sensitivity analysis

**Fig. 4:**
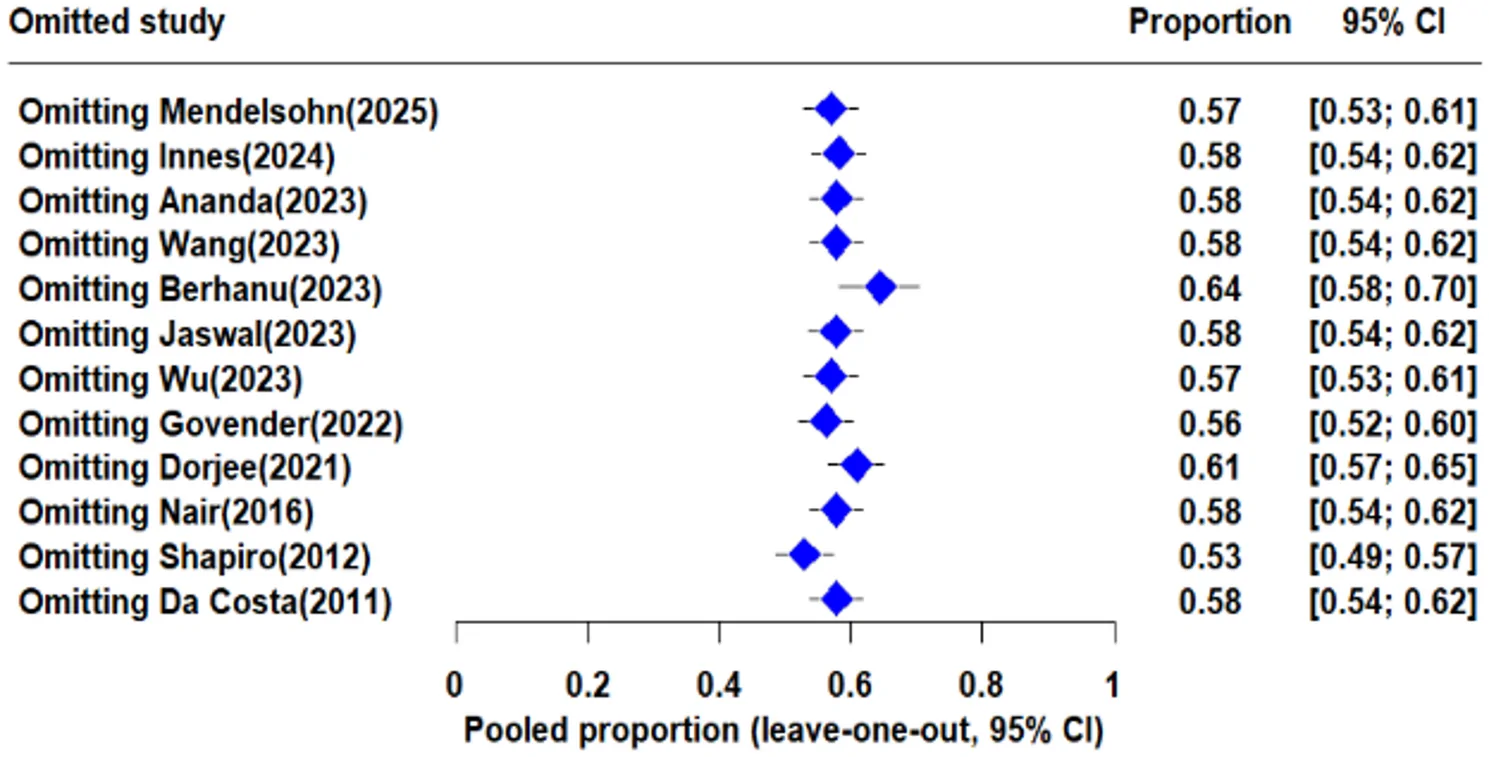
Leave-One-Out sensitivity analysis for proportion estimates among adolescents and young adults (AYAs) with TB

Sensitivity analyses using a leave-one-out method indicated that no single study unduly influenced the pooled prevalence estimates in the meta-analysis. In the prevalence meta-analysis, the pooled prevalence remained 1% (95% CI ~ 1%–1%) regardless of which study was excluded, with I² consistently ~ 97%, indicating that heterogeneity reflected broad between-study variability rather than a single outlier (Supplementary Figure S22). In the proportion meta-analysis, the pooled proportion ranged from 53% to 64% when studies were omitted (Fig. 4). We also excluded studies from Europe and America, which did not change our results significantly. Publication bias was not significant, as neither Egger’s nor Begg’s tests indicated funnel asymmetry, and trim-and-fill produced only a negligible change in the pooled estimate (Supplementary Figure S23).

## Discussion

To the best of our knowledge, this is the first systematic review to estimate the prevalence of asymptomatic (subclinical) TB in AYAs and the proportion of TB in AYAs with TB that is asymptomatic. We found that approximately 1% of screened AYAs in community, household, and active case-finding settings have bacteriologically confirmed TB despite reporting no symptoms across countries in Asia and Africa. In addition, we estimated that the proportion of asymptomatic TB out of all bacteriologically confirmed pulmonary TB in AYAs was 62%, with a pooled estimate of 73% among studies with low risk of bias.

Our prevalence estimate was higher than findings from general population studies reporting a pooled prevalence of asymptomatic TB of 0.2% (95% CI: 0.2%-0.3%) among individuals aged ≥ 15 years in 25 studies [34]. Prior pooled estimates of asymptomatic TB proportion among the general population ranged from 23% to 83% [30–35]. An individual participant meta-analysis by Stuck et al. [36] estimated a proportion of 39.4% among individuals aged 15–24 years in four African countries, which is lower than our pooled estimate of 62%. All these studies applied the same definition for asymptomatic TB as our study, but some studies included different screening approaches for identifying microbiologically confirmed TB. The differences in region, settings and the way they screened participants might explain differences in reported estimates. Regional and epidemiological context, high HIV-burden settings and differences in screening algorithms influence estimates of asymptomatic TB.

Importantly, our results showed no major difference in the prevalence and proportion of asymptomatic TB between adolescents (10–19 years) and young adults (20–24 years), with both groups showing 1% prevalence and 50–60% proportion of asymptomatic TB. Given the small number of studies, we caution against overinterpretation of findings between the age categories. In addition, our study showed that population-level prevalence estimates were not influenced by geographic regions. Also, no regional differences were found for the proportion of asymptomatic TB. This finding is inconsistent with Mahmoudi et al.‘s [34] meta-analysis, which found that the proportion of all TB that is asymptomatic in the general population was higher in Asia compared to Africa, 60% vs. 44%, respectively. Emery et al. [37] also found after adjusting for disease duration, subclinical TB has a relative infectiousness of 1.93 (0.62–6.18) compared to clinical TB. Overall, subclinical TB accounts for an estimated 68% (27–92%) of global Mtb transmission. This might be because of higher numbers of PLHIV in African countries included in Mahmoudi’s study. Several studies have shown that PLHIV may progress more quickly from asymptomatic to symptomatic TB, which shortens the period during which asymptomatic disease can be detected [20, 38–43]. While this evidence is important, our review did not include enough studies with HIV-disaggregated data to explore this relationship.

The papers that were included in our review employed different strategies that enable detection of asymptomatic TB. Most included studies applied multistep ACF algorithms, typically beginning with an initial step such as a symptom questionnaire [18, 23, 27] or chest radiography [22, 25] followed by confirmatory microbiological testing (Xpert MTB/RIF, culture, or both). Evidence from multiple settings shows that approaches combining symptom screening with chest radiography and molecular diagnostics substantially increase detection rates and allow for the earlier identification of subclinical or asymptomatic TB [1–5]. Conducting these strategies in parallel overcomes a major shortcoming of symptom-based ACF alone [44, 45], reducing delays in diagnosis and treatment.

The sensitivity analysis showed that excluding Berhanu [32] or Shapiro [22] decreased heterogeneity, especially for the proportion outcome. A facility-based targeted universal testing with systematic symptom screening, and then sputum microbiology, was done by Berhanu. The targeted universal testing algorithm used in this study offered all primary care attendees with at least one risk factor, including HIV infection, previous TB exposure, and a history of diagnosed TB in the past two years, a test regardless of symptoms. While a symptom-independent method was used for testing, the study setting of clinics may have biased the estimate towards increased proportion of symptomatic TB. Shapiro et al. [22] implemented community-based household contact tracing among people with TB, providing universal symptom screening and sputum culture for all household members, regardless of symptoms or risk factors, together with optional HIV testing offered as part of the screening process. In that study, 89% of individuals with TB did not report symptoms at the time of the household visit, and 93% of those with TB were diagnosed by sputum culture alone. These findings contrast with most other included studies, which relied on symptom-triggered algorithms or conventional programmatic approaches, often conducted in low-HIV prevalence settings. The high proportion of asymptomatic TB may also be due to higher proportion of people with early-stage TB compared to other studies, which may have been a result of the contact-tracing activities identifying TB early after infection. Generally, a high TB/HIV burden setting, and mode of strategies and screening, might influence the detection of asymptomatic TB.

Overall, this paper highlights a critical gap in how TB data has been historically categorized. Most past studies and surveillance reports did not separately categorize “adolescents” or “young adults” in accordance with WHO definitions. Even reporting classifications used by the WHO have historically omitted the adolescent age group by categorizing TB as occurring in “children” (0–14 years) or “adults” (15 years and older) [2]. This practice runs against WHO’s recommended age breakdowns and has resulted in a dearth of evidence specific for this age group. Fortunately, this gap is starting to be addressed. Global policy has shifted towards better age-disaggregated data for TB. Since 2020, WHO has requested countries to report TB in finer age bands, including 10–14 years, 15–19 years and 20–24 years as separate categories. This change, strongly encouraged in updated WHO surveillance guidance, aims to illuminate the TB burden in AYAs that was previously obscured. Moreover, the global TB community has recognized this issue: the 2018 *Roadmap Towards Ending TB in Children and Adolescents* explicitly highlighted the lack of adolescent-specific data and called for improved reporting and research in this group [46].

The AYA population is a demographically significant group for TB epidemiology but, until now, a neglected population in surveillance and policy. From a global perspective, TB incidence increases rapidly during adolescence, reaching the highest levels in early adulthood, including among AYAs [4, 46, 47]. This increased vulnerability in this population group may be due to increased susceptibility during puberty [48] combined with an expanding group of social contacts that involve, but are not limited to, school, work, public transportation, as well as other gathering sites focusing on being major transmission channels for all these groups [49, 50]. These observations support the need for more granular age-disaggregated reporting (e.g., 10–14, 15–19 and 20–24 years) and for targeted screening and diagnostic approaches in the settings where young people most commonly interact. AYAs-focused screening strategies should therefore consider non symptoms approaches in high-risk populations and settings where young people commonly interact, particularly among household contacts and in schools, universities, workplaces, congregate settings, and high-burden communities. Based on available resources and local settings, these strategies may include chest radiography followed by rapid molecular testing, or direct molecular testing using sputum or appropriate non-sputum specimens when sputum collection is difficult. For AYAs, the main benefits of these approaches would be earlier identification of missed TB, reduced diagnostic delays, rapid treatment initiation, and prevention of onward transmission within their broad social networks. Although the WHO continues to update recommendations for TB screening and diagnosis, clearer operational guidance is still needed on how symptom-agnostic, radiographic, and molecular screening approaches should be adapted and implemented specifically for AYA populations [20–22, 26, 32].

There were several limitations in this study. First, one study included AYAs (10–24 years old) alongside other participants and did not report results disaggregated specifically for the AYAs subgroup. More broadly, some studies combined adolescents and young adults into relatively wide age categories, limiting our ability to generate separate pooled estimates for the 10–14, 15–19, and 20–24-year age groups. This is important because TB risk, patterns of social contact, and access to health services may differ across these developmental stages. Second, limited information was available on asymptomatic TB among people living with HIV. Consequently, we were unable to stratify the pooled estimates by HIV status, degree of immunosuppression, antiretroviral therapy use, or other HIV-related clinical characteristics. Because HIV may affect TB presentation, symptom expression, bacillary burden, and the performance of available diagnostic tests, the absence of HIV-stratified data limits the interpretation and generalizability of our findings for AYAs living with HIV. Considerable heterogeneity was observed among the studies included. The studies differed in geographic region, epidemiological context, study setting, population characteristics, age distribution, TB exposure risk, recruitment strategy, and study design. Subgroup analyses, meta-regression, sensitivity analyses, and random-effects and fixed-effect models were conducted in order to explore sources of heterogeneity. Furthermore, the number of studies included in some subgroup and meta-regression analyses was small, which limited the generalizability. Another limitation is that the definition of asymptomatic bacteriologically confirmed TB generally depended on sputum-based microbiological testing. This approach may underestimate the true estimate among younger adolescents who are unable to produce an adequate sputum specimen. Differences in specimen type, specimen quality, number of specimens collected, and diagnostic methods, including smear microscopy, culture, and molecular testing, may also have affected case detection. Also, the screening and diagnostic algorithms varied substantially among studies. Some studies performed universal bacteriological testing regardless of symptoms, whereas others used conditional approaches in which bacteriological testing was conducted only among participants with symptoms, abnormal chest radiography, known TB exposure, or other initial screening findings. Conditional algorithms may systematically miss asymptomatic individuals who do not meet the criteria for further testing. Lastly, symptom assessment was not standardized across the included studies. Studies differed in the symptoms evaluated, the number and duration of symptoms required, the recall periods used, and whether symptoms were self-reported, caregiver-reported, or assessed by research or clinical staff.

## Conclusion

Our study highlighted that the overall prevalence of asymptomatic TB among AYAs may be high (about 1%) and approximately two-thirds of bacteriologically confirmed TB in this age group may be asymptomatic. This pattern was consistent across age subgroups and screening approaches. The result showed that age-disaggregated surveillance and broader use of radiographic and molecular tools may be needed for detecting early or asymptomatic TB in AYAs. However, these findings should be interpreted cautiously because of the considerable heterogeneity across studies, variation in screening and diagnostic algorithms, lack of standardized symptom assessment, and the small number of studies available for some subgroup analyses. Despite these limitations, the findings support more granular age-disaggregated surveillance and further evaluation of radiographic and molecular tools for detecting early or asymptomatic TB among AYAs. Future studies should use standardized definitions of asymptomatic TB and consistent age categories, clearly report symptom-assessment and diagnostic procedures, and provide results stratified by HIV status and other important clinical characteristics.

## Supplementary Information

Below is the link to the electronic supplementary material.

Supplementary Material 1

## Data Availability

The datasets generated and/or analyzed during the current study, including extracted data and analytic code, are available from the corresponding author upon reasonable request.

## Abbreviations

ACF: Active Case Finding
AI: Artificial Intelligence
AYAs: Adolescents and Young Adults
CAD: Computer–Aided Detection
CDC: Centers for Disease Control and Prevention
CI: Confidence Interval
CINAHL: Cumulative Index to Nursing and Allied Health Literature
CXR: Chest X–ray
GLMM: Generalized Linear Mixed Model
HIV: Human Immunodeficiency Virus
IGRA: Interferon–Gamma Release Assay
JBI: Joanna Briggs Institute
MeSH: Medical Subject Headings
Mtb: Mycobacterium tuberculosis
NAAT: Nucleic Acid Amplification Test
OTAD: Open Access Theses and Dissertations
PLHIV: People Living with HIV
PRISMA: Preferred Reporting Items for Systematic Reviews and Meta–Analyses
PROSPERO: International Prospective Register of Systematic Reviews
PTB: Pulmonary Tuberculosis
Q test: Cochran’s Q test for heterogeneity
TB: Tuberculosis
TST: Tuberculin Skin Test
WHO: World Health Organization
Xpert MTB/RIF: Xpert Mycobacterium tuberculosis/Rifampicin assay
