# Supplementary material for: The prevalence and proportion of asymptomatic tuberculosis among adolescents and young adults: a systematic review and meta-analysis
Source: BMC Infect Dis. 2026 Aug 4;26:1919. doi: 10.1186/s12879-026-14156-y (PMC13726186; doi:10.1186/s12879-026-14156-y)

The prevalence and proportion of asymptomatic tuberculosis among adolescents and young adults: A systematic review and meta-analysis

Heidari ME[1], Ngo N[1], Modongo C[2], Malik AA[3,4], Jaswal MR[5], Shapiro AE[6,7], Rebecca H Berhanu[8,9], Neil A Martinson[10,11], Lebina L[12], Niemann S[13-15] Seddon JA[16,17], Shin SS[1]*

1. Sue & Bill Gross School of Nursing, University of California Irvine, Irvine, CA, USA; 2. Victus Global Botswana Organization, Gaborone, Botswana; 3. O'Donnell School of Public Health, UT Southwestern Medical Center, Dallas, TX, USA; 4. IRD Global, Singapore; 5. Baitussalam Welfare Trust, Pakistan; 6.Department of Global Health, University of Washington, Seattle, Washington, USA; 7.Division of Allergy and Infectious Diseases, Department of Medicine, University of Washington, Seattle, Washington, USA; 8.University of the Witwatersrand, Johannesburg, South Africa; 9.Department of Medicine, Division of Infectious Diseases, Vanderbilt University Medical Center, Nashville, TN, USA; 10.Perinatal HIV Research Unit, University of the Witwatersrand, Johannesburg; 11.Johns Hopkins University Center for TB Research, Baltimore; 12.Africa Health Research Institute, Clinical Trials Unit, KwaZulu-Natal, South Africa; 13.Research Center Borstel, Leibniz Lung Center, Borstel, Germany; 14. German Center for Infection Research, Partner Site Hamburg-Lübeck-Borstel-Riems, Borstel, Germany, 15. 9 EPHE, PSL University, and Institut de Systématique, Evolution, Biodiversité, ISYEB, Muséum national d'Histoire naturelle, CNRS, Sorbonne Université, EPHE, Université des Antilles, Paris, France; 16. Desmond Tutu TB Centre, Department of Pediatrics and Child Health, Stellenbosch University, Cape Town, South Africa; 17. Department of Infectious Disease, Imperial College London, London, United Kingdom

Table of Contents

Supplementary Tables

- Supplementary Table S1. Search strategy and keywords used across databases
- Supplementary Table S2. JBI Critical Appraisal Checklist for studies reporting prevalence data
- Supplementary Table S3. Risk of bias assessment
- Supplementary Table S4. Meta-regression of asymptomatic TB prevalence with mean age as a moderator
- Supplementary Table S5. Meta-regression of asymptomatic TB prevalence with region as a moderator
- Supplementary Table S6. Multivariable meta-regression of prevalence by mean age and region
- Supplementary Table S7. Meta-regression of asymptomatic TB proportion with mean age as a moderator
- Supplementary Table S8. Meta-regression of asymptomatic TB proportion with region as a moderator
- Supplementary Table S9. Multivariable meta-regression of proportion by mean age and region

Supplementary Figures

- Supplementary Figure S1. Pooled prevalence of asymptomatic TB in studies with low risk of bias
- Supplementary Figure S2. Pooled proportion of asymptomatic TB in studies with low risk of bias
- Supplementary Figure S3. Pooled prevalence of asymptomatic TB in studies with moderate risk of bias
- Supplementary Figure S4. Pooled proportion of asymptomatic TB in studies with moderate risk of bias

- Supplementary Figure S5. Pooled proportion of asymptomatic TB in studies with high risk of bias
- Supplementary Figure S6. Pooled prevalence of asymptomatic TB among adolescents
- Supplementary Figure S7. Pooled prevalence of asymptomatic TB among young adults
- Supplementary Figure S8. Pooled prevalence of asymptomatic TB in studies published in the last 5 years
- Supplementary Figure S9. Pooled prevalence of asymptomatic TB among studies in Africa
- Supplementary Figure S10. Pooled prevalence of asymptomatic TB among studies in Asia
- Supplementary Figure S11. Pooled proportion of asymptomatic TB among studies published in the last 5 years
- Supplementary Figure S12. Pooled proportion of asymptomatic TB among adolescents
- Supplementary Figure S13. Pooled proportion of asymptomatic TB among young adults
- Supplementary Figure S14. Pooled proportion of asymptomatic TB among studies in Africa
- Supplementary Figure S15. Pooled proportion of asymptomatic TB among studies in Asia
- Supplementary Figure S16. Pooled prevalence of asymptomatic TB among studies that applied universal bacteriological testing
- Supplementary Figure S17. Pooled prevalence of asymptomatic TB among studies that applied conditional bacteriological testing
- Supplementary Figure S18. Pooled proportion of asymptomatic TB among studies that applied universal bacteriological testing
- Supplementary Figure S19. Pooled proportion of asymptomatic TB among studies that applied conditional bacteriological testing
- Supplementary Figure S20. Pooled prevalence of asymptomatic TB among studies that included household contact in the screening process
- Supplementary Figure S21. Pooled proportion of asymptomatic TB among studies that included household contact in the screening process
- Supplementary Figure S22. Leave-one-out analysis for prevalence estimation

- Supplementary Figure S23. Funnel plots of (a) prevalence of TB in AYAs and (b) proportion of asymptomatic TB among AYAs with TB

Sensitivity search strategy

- Sensitivity search strategy with meta-analysis

Table S1. Search strategy and keywords used across databases

**Databases**	**Search strategy**
PubMed	(Tuberculosis, Latent[MeSH] OR "latent tuberculosis infection" OR LTBI OR "asymptomatic tuberculosis" OR "Subclinical tuberculosis ") AND ("Child"[MeSH] OR "Adolescent"[MeSH] OR child* OR adolescent* OR minor* OR youth OR "pediatric population" OR "school-age children" OR "School") AND ("Prevalence"[MeSH] OR prevalence OR incidence OR epidemiology)
Web of science	(((ALL=(Tuberculosis, Latent OR latent tuberculosis infection OR LTBI OR asymptomatic tuberculosis OR Subclinical tuberculosis)) AND ALL=(Child OR Adolescent OR child OR adolescent OR minor OR youth OR pediatric population OR school-age children OR School)) AND ALL=(Prevalence OR prevalence OR incidence OR epidemiology))

Scopus	(TITLE-ABS-KEY (asymptomatic AND tuberculosis OR subclinical AND tuberculosis) AND ALL (child OR adolescent OR teenager OR child OR adolescent OR minor OR youth OR pediatric AND population OR school-age AND children OR school OR college OR university) AND ALL (prevalence OR prevalence OR incidence OR epidemiology OR outbreak))
CINAHL	TX (tuberculosis or tb) AND TX (school OR Child OR pediatric OR Children OR college OR University)

Table S2: JBI Critical Appraisal Checklist for studies reporting prevalence data

Reviewer_______________________________________

Date__________________________

Author____________________________________Year_________ Record Number_________

	Yes	No	Unclear	Not applicable
1. Was the sample frame appropriate to address the target population?	□	□	□	□
2. Were study participants sampled in an appropriate way?	□	□	□	□
3. Was the sample size adequate?	□	□	□	□
4. Were the study subjects and the setting described in detail?	□	□	□	□
5. Was the data analysis conducted with sufficient coverage of the identified sample?	□	□	□	□
6. Were valid methods used for the identification of the condition?	□	□	□	□

**7. Was the condition measured in a standard, reliable way for all participants?**	□	□	□	□
**8. Was there appropriate statistical analysis?**	□	□	□	□
**9. Was the response rate adequate, and if not, was the low response rate managed appropriately?**	□	□	□	□

Overall appraisal: **Include** □ **Exclude** □ **Seek further info** □

Comments (Including reason for exclusion)

____

____

____

Table S3. Risk of bias

First Author of Study (Year)	Final judgment
Nur Rahmi Ananda (2023)	**Low**
Kunchok Dorjee (2021)	**Low**
K. Gunasekera (2020)	**Low**
Abilash Nair (2016)	**Low**
B. Odume (2024)	**Low**
Zhongdong Wang (2023)	**High**
Naomi Carter (2023)	**Low**
Simon C Mendelsohn (2025)	**Low**
Costa et al (2011)	**Moderate**
Qian et al (2023)	**Low**
Simon C. Mendelsohn (2022)	**Moderate**
Rebecca H. Berhanu (2023)	**Moderate**

Maria Rauf Jaswal (2023)	**Low**
Anh L Innes (2024)	**High**
Adrienne E. Shapiro (2012)	**Low**
Indira Govender (2022)	**Low**

Table S4. Meta-regression of asymptomatic TB prevalence with mean age as a moderator

Moderator	Number of included studies	Estimate (β)	SE	z-value	p-value	95% CI (lower)	95% CI (upper)
Intercept		–3.56	3.06	–1.16	0.24	–9.56	2.44
Mean age	5	–0.02	0.16	–0.14	0.88	–0.35	0.30
Model output: $\tau^2 = 1.12$; $I^2 = 95.32\%$; $QM\ (df = 1) = 0.02$, $p = 0.88$							

Table S5. Meta-regression of asymptomatic TB prevalence with region as a moderator

Moderator[1]	Number of included studies	Estimate (β)	SE	z-value	p-value	95% CI (lower)	95% CI (upper)
Intercept		–3.94	0.35	–11.03	<0.001	–4.64	–3.24
Region Asia	3	–2.01	0.78	–2.55	<0.01	–3.56	–0.46
Model output: $\tau^2 = 0.91$; $I^2 = 96.60\%$; $QM\ (df = 1) = 6.51$, $p = 0.01$							

1: Africa with 8 studies as reference category

Table S6. Multivariable meta-regression of prevalence by mean age and region

Moderator	Estimate (β)	SE	z-value	p-value	95% CI (lower)	95% CI (upper)
Intercept	-1.51	3.40	-0.44	0.65	-8.18	5.15
Mean age	–0.12	0.17	–0.67	0.49	–0.47	0.22
Region Asia	–1.40	1.32	–1.06	0.28	–3.99	1.18
Model output: $\tau^2 = 0.85$ $I^2 = 92.73\%$; *QM (df = 2) = 6.35, p =0.04*						

Table S7. Meta-regression of asymptomatic TB proportion with mean age as a moderator

Moderator	Number of included studies	Estimate (β)	SE	z-value	p-value	95% CI (lower)	95% CI (upper)
Intercept		8.42	1.39	6.04	0.001	5.68	11.15
Mean age	5	–0.38	0.06	–5.83	0.001	–0.51	-0.25
Model output: *$\tau^2 = 0$; $I^2 = 0$; QM (df = 1) = 34.10, p = 0.001*							

Table S8. Meta-regression of asymptomatic TB proportion with region as a moderator

Moderator	Number of included studies	Estimate (β)	SE	z-value	p-value	95% CI (lower)	95% CI (upper)
Intercept		1.71	0.59	2.90	0.003	0.55	2.87
Region Asia	7	–1.86	0.77	-2.41	0.01	–3.38	-0.35
Model output: $\tau^2 = 0.99$; $I^2 = 79.16\%$; $QM\ (df = 1) = 5.84$, $p = 0.01$							

Table S9. Multivariable meta-regression of prevalence by mean age and region

Moderator	Estimate (β)	SE	z-value	p-value	95% CI (lower)	95% CI (upper)
Intercept	8.48	1.52	5.55	<0.001	5.49	11.48
Mean age	–0.38	0.07	–5.37	<0.001	-0.53	-0.24
Region Asia	-0.24	1.13	-0.21	0.82	-2.47	1.98
Model output: $\tau^2 = 0$; $I^2 = 0\%$; $QM\ (df = 2) = 38.84$, $p < 0.001$						

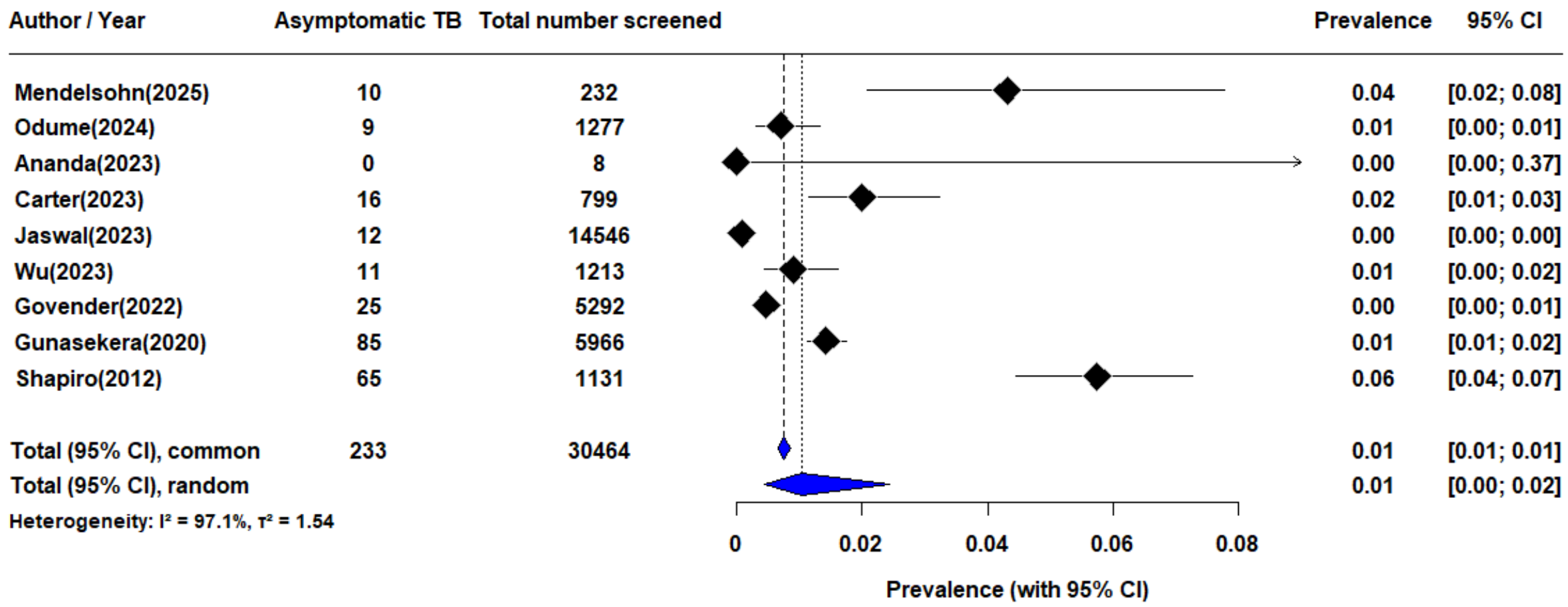

Figure S1. Pooled prevalence of asymptomatic TB in studies with low risk of bias

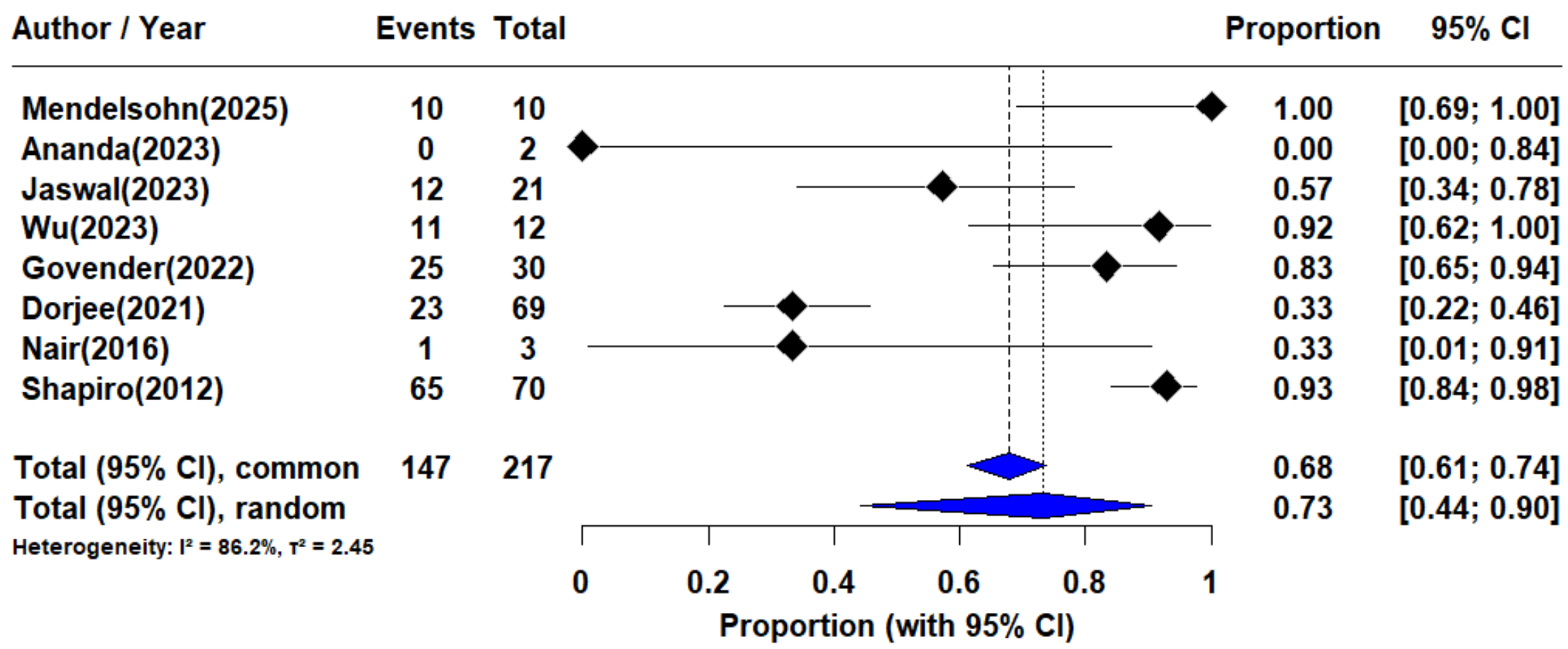

Figure S2. Pooled proportion of asymptomatic TB in studies with low risk of bias

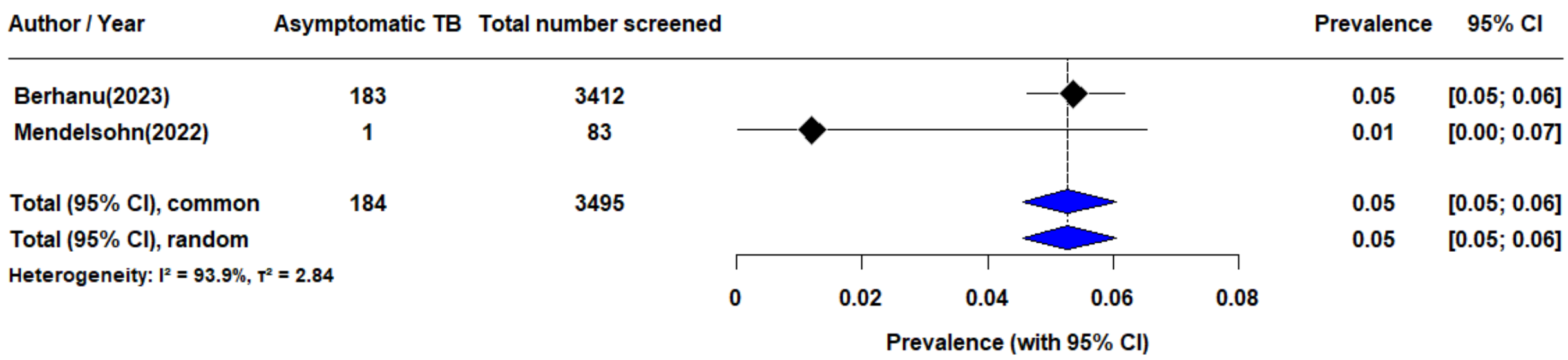

Figure S3. Pooled prevalence of asymptomatic TB in studies with moderate risk of bias

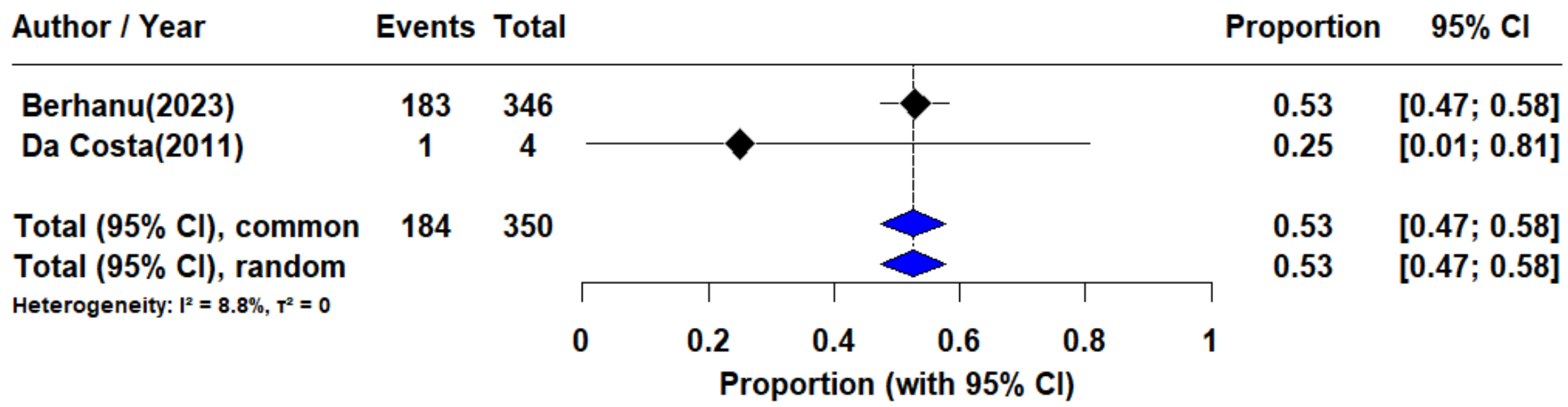

Figure S4. Pooled proportion of asymptomatic TB in studies with moderate risk of bias

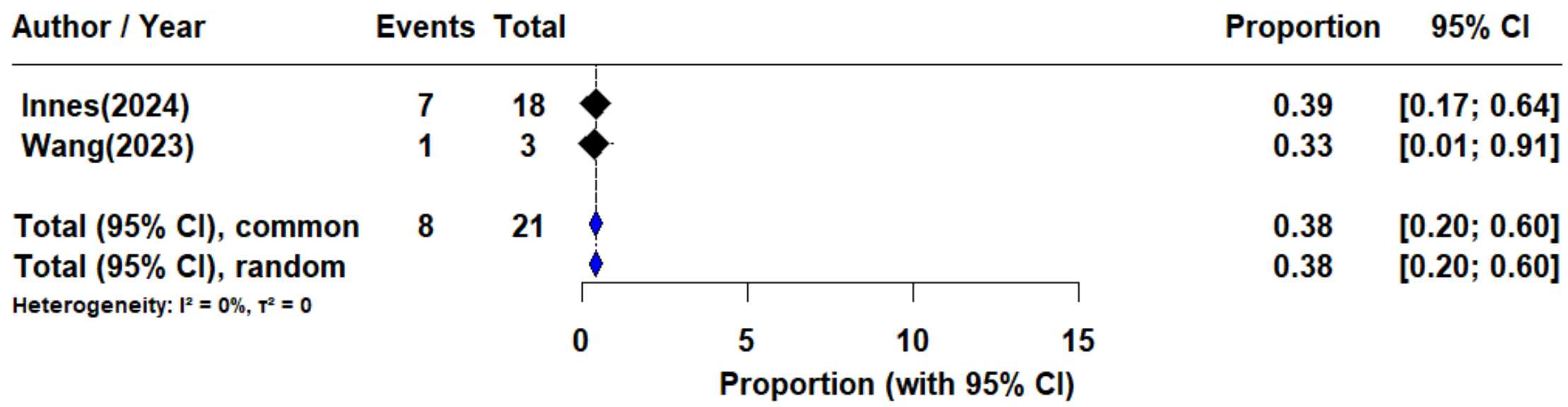

Figure S5. Pooled proportion of asymptomatic TB in studies with high risk of bias

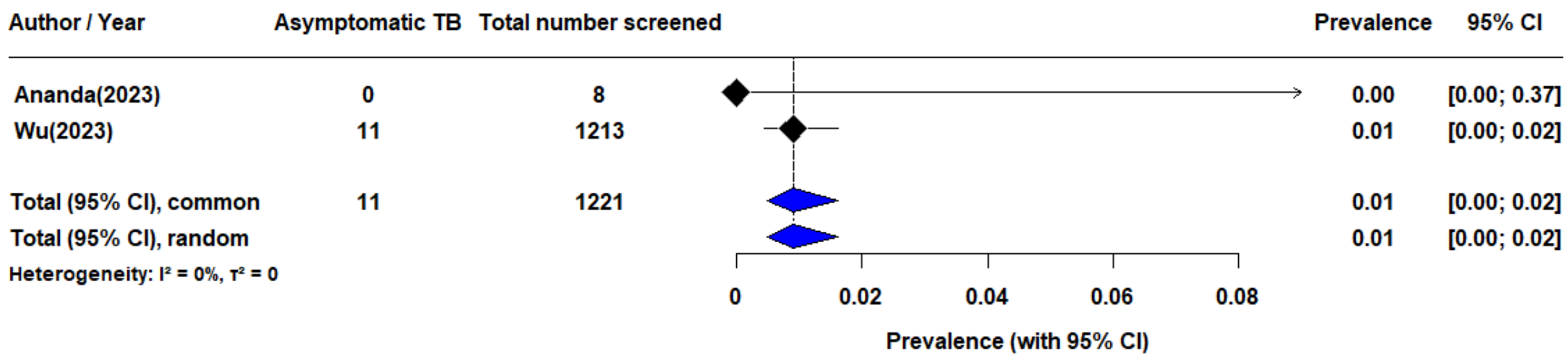

Figure S6. Pooled prevalence of asymptomatic TB among adolescents

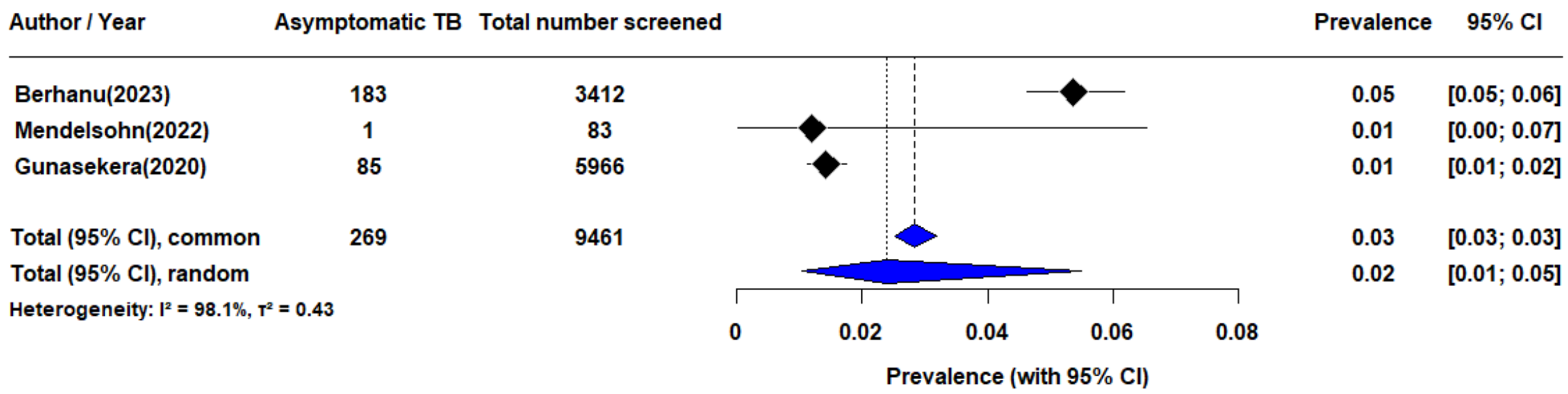

Figure S7. Pooled prevalence of asymptomatic TB among young adults

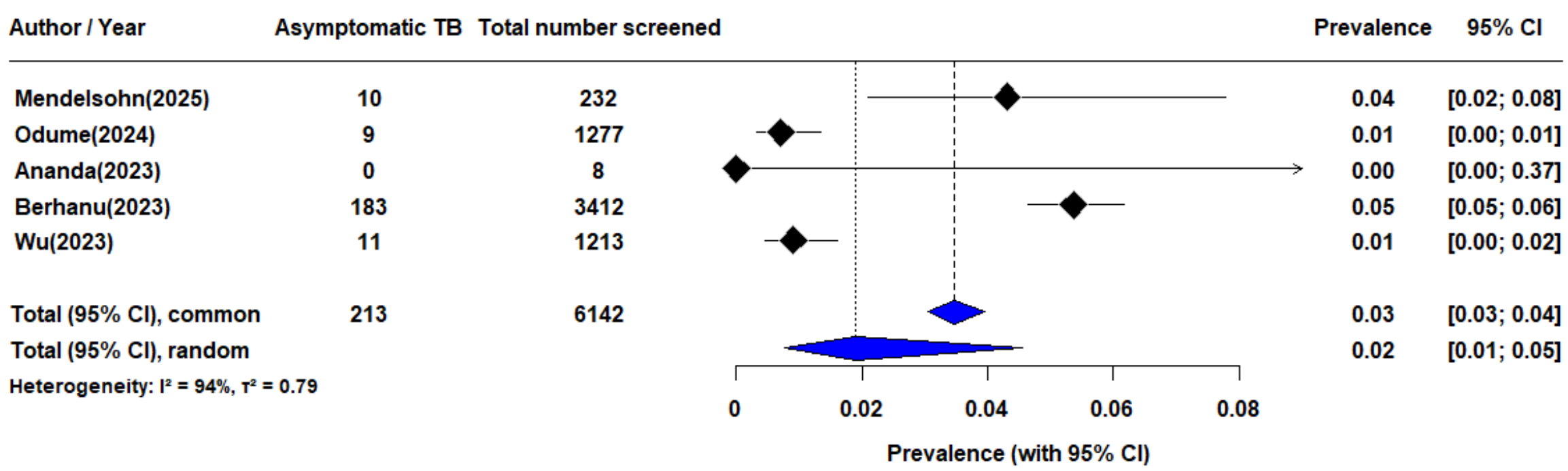

Figure S8. Pooled prevalence of asymptomatic TB in studies published in the last 5 years

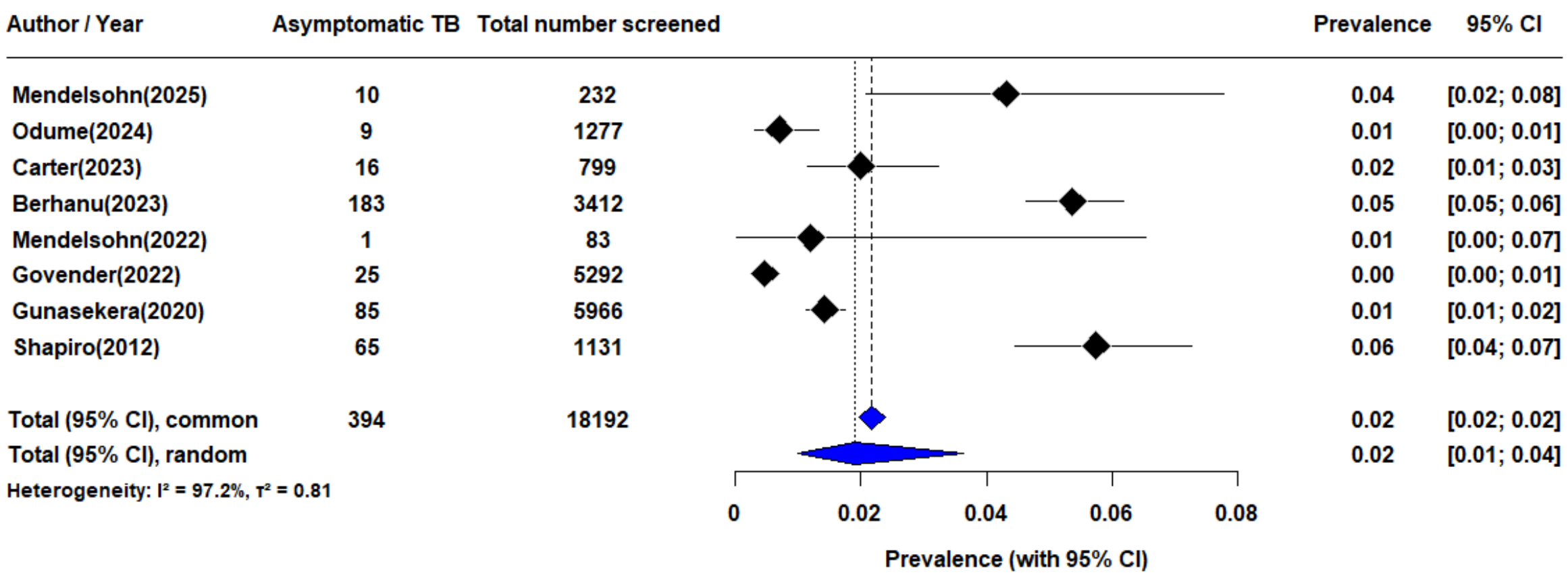

Figure S9. Pooled prevalence of asymptomatic TB among studies in Africa

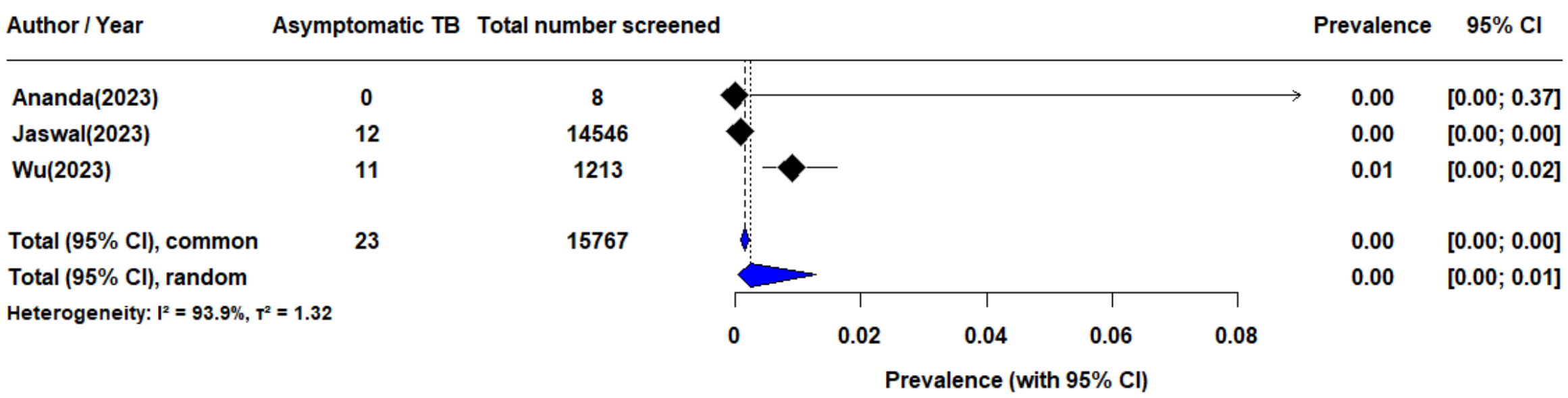

Figure S10. Pooled prevalence of asymptomatic TB among studies in Asia.

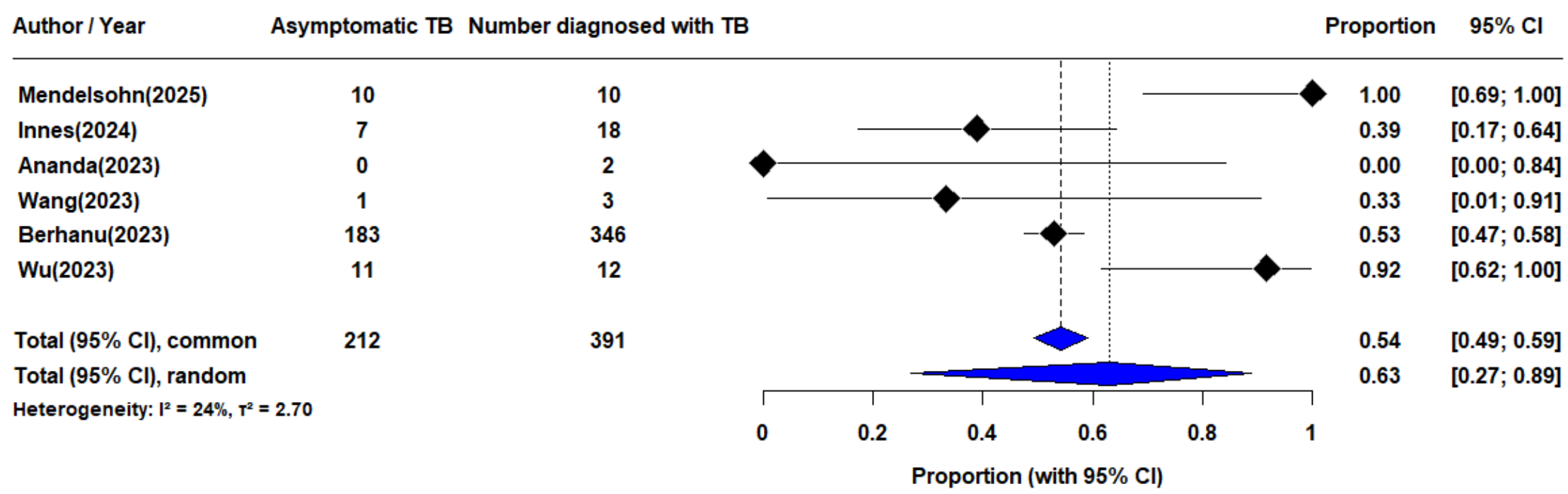

Figure S11. Pooled proportion of asymptomatic TB among studies published in the last 5 years

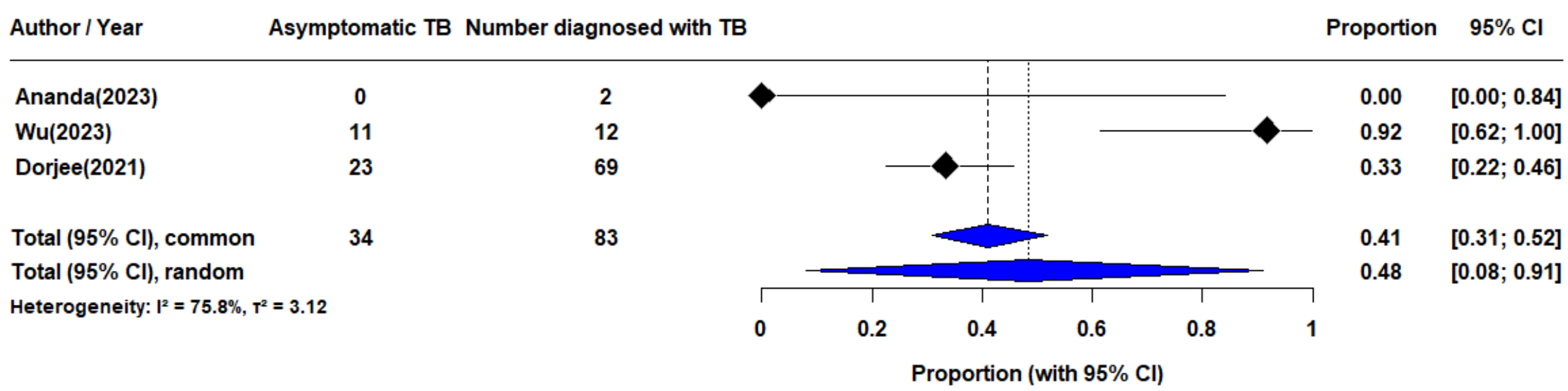

Figure S12. Pooled proportion of asymptomatic TB among adolescents

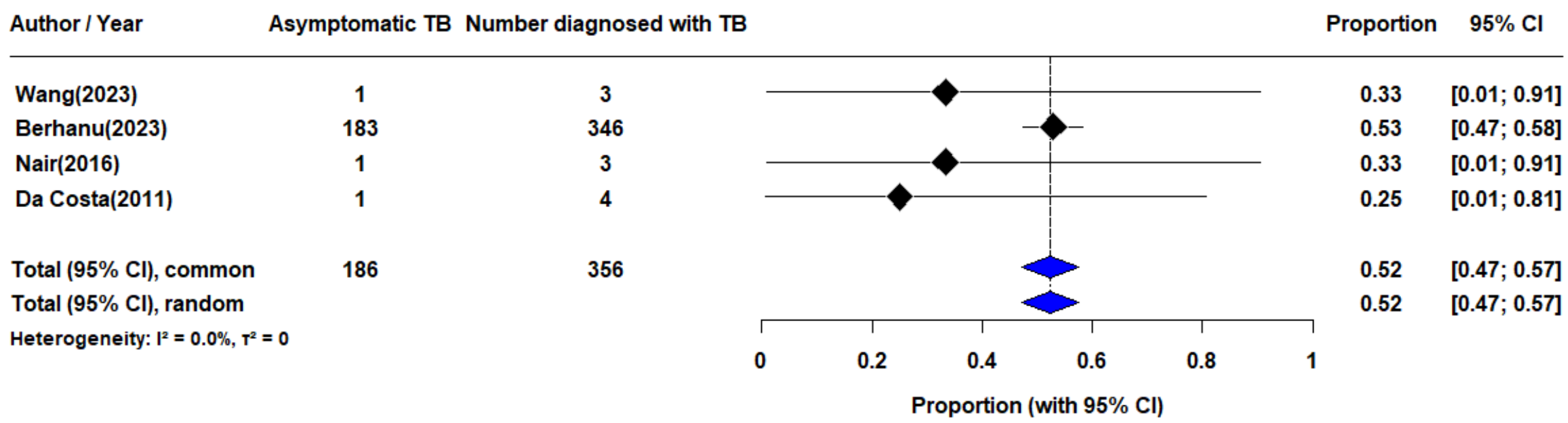

Figure S13. Pooled proportion of asymptomatic TB among young adults

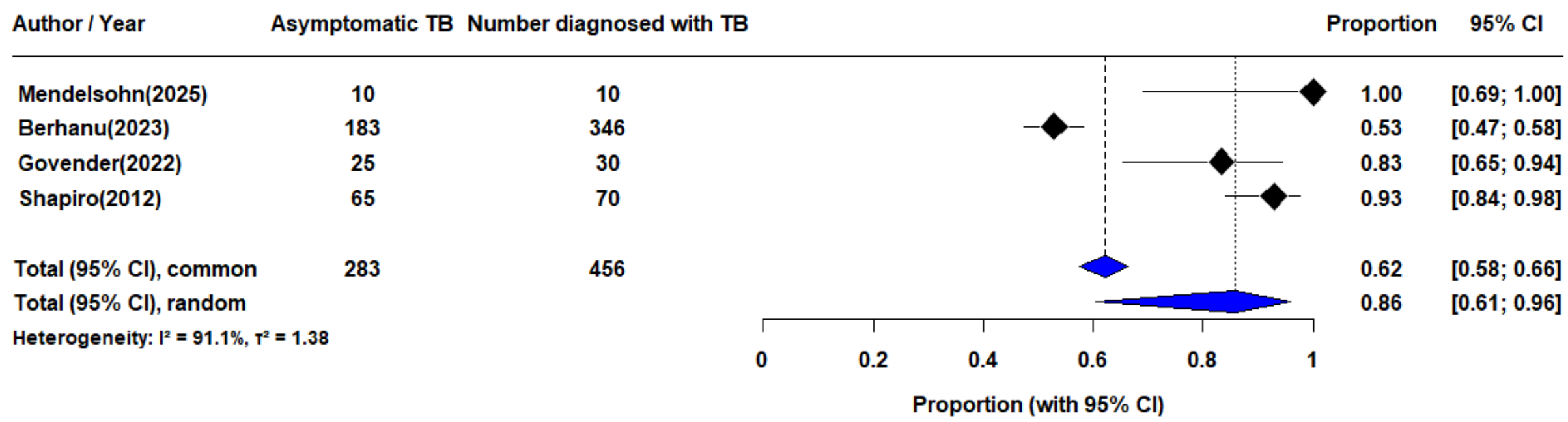

Figure S14. Pooled proportion of asymptomatic TB among studies in Africa

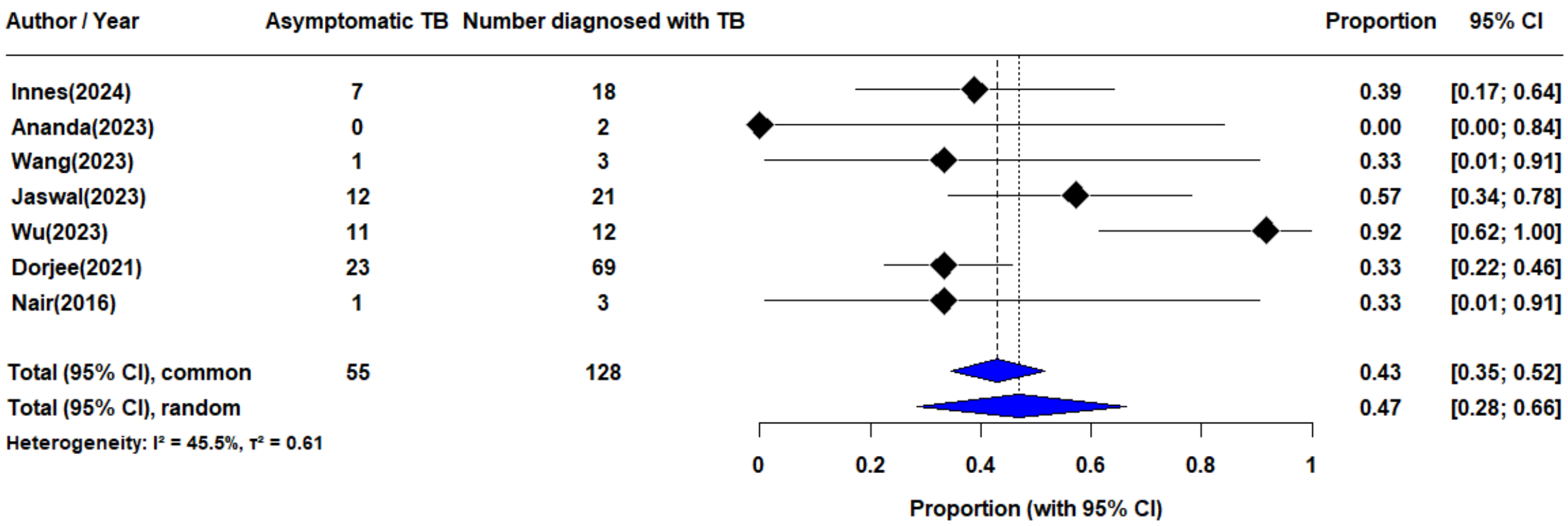

Figure S15. Pooled proportion of asymptomatic TB among studies in Asia

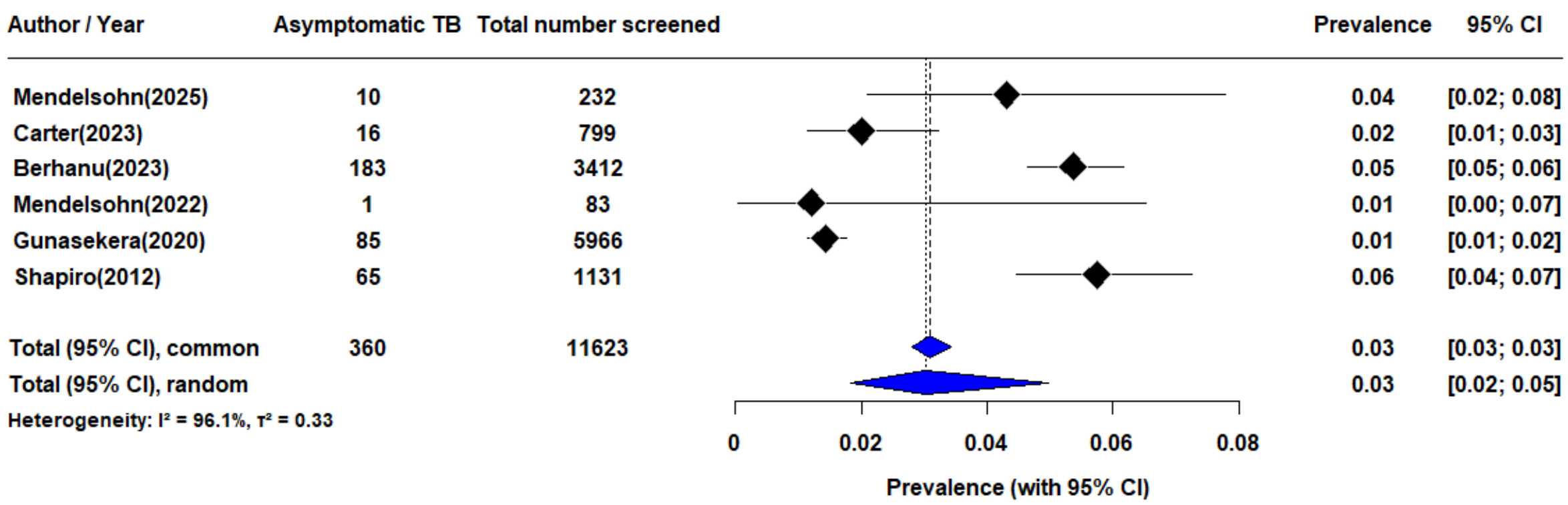

Figure S16. Pooled prevalence of asymptomatic TB among studies that applied universal bacteriological testing

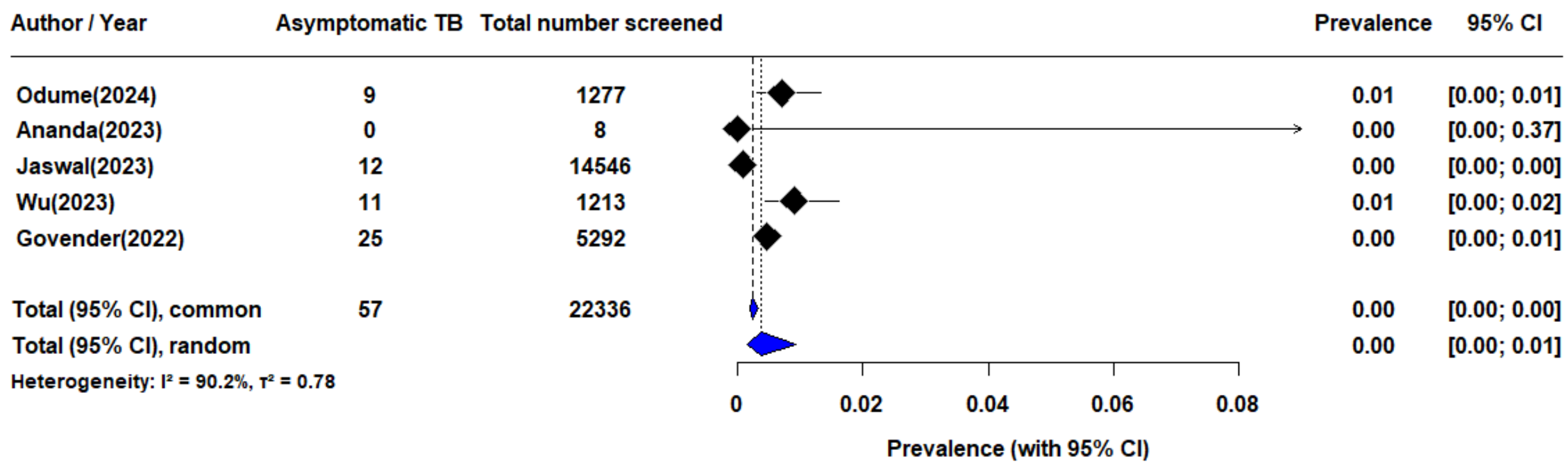

Figure S17. Pooled prevalence of asymptomatic TB among studies that applied conditional bacteriological testing

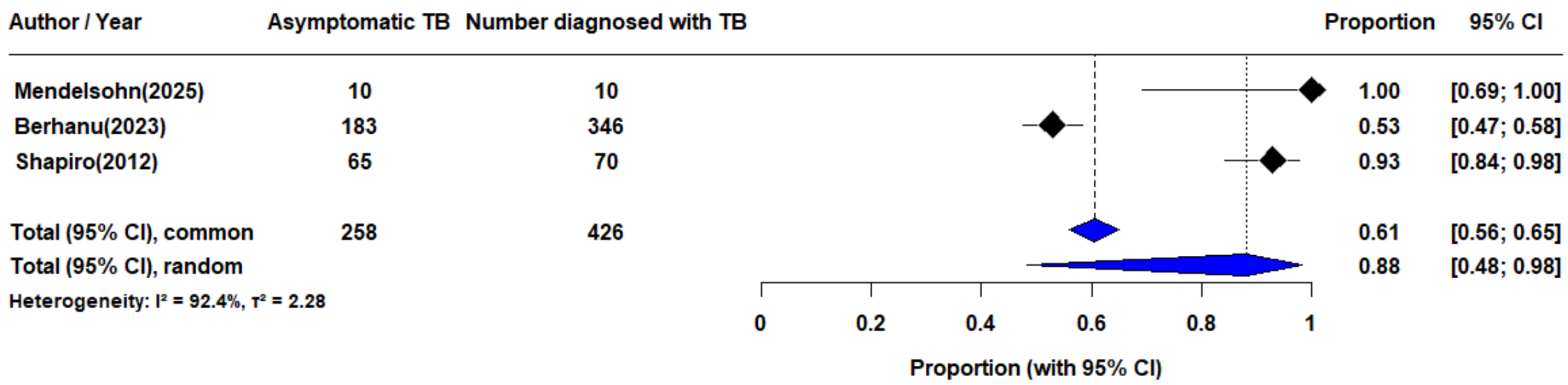

Figure S18. Pooled proportion of asymptomatic TB among studies that applied universal bacteriological testing

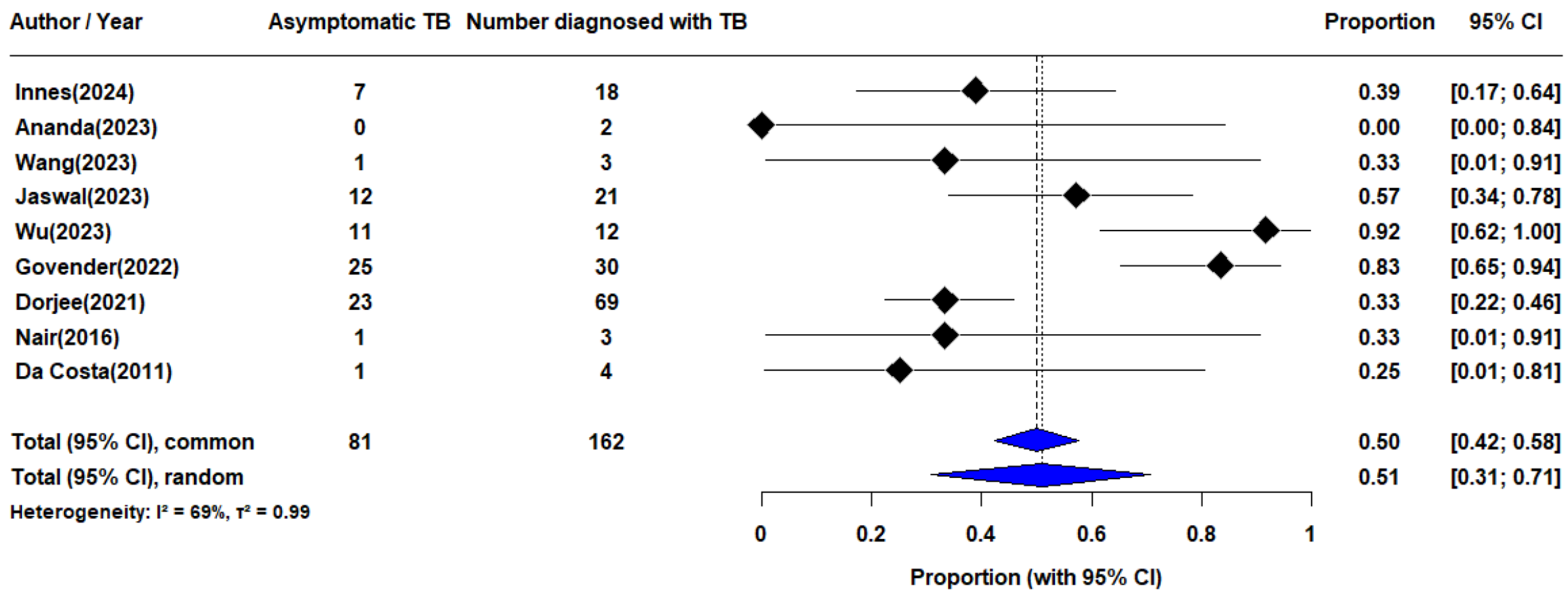

Figure S19. Pooled proportion of asymptomatic TB among studies that applied conditional bacteriological testing

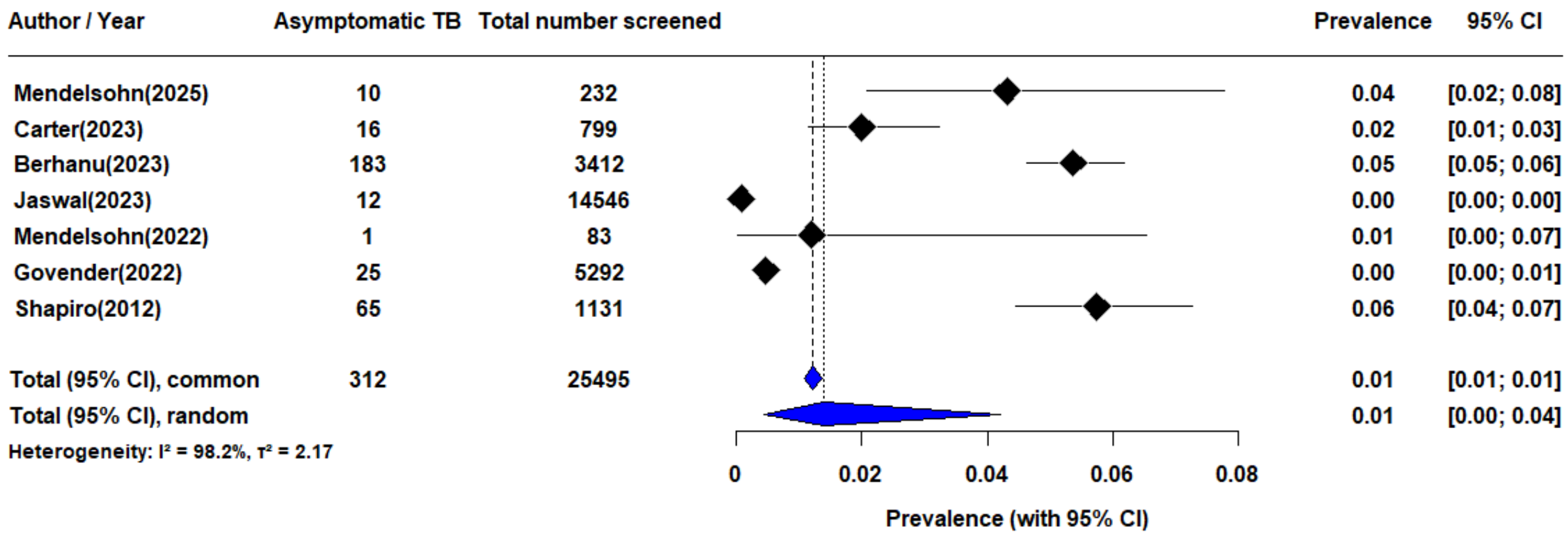

Figure S20. Pooled prevalence of asymptomatic TB among studies that included household contact in the screening process

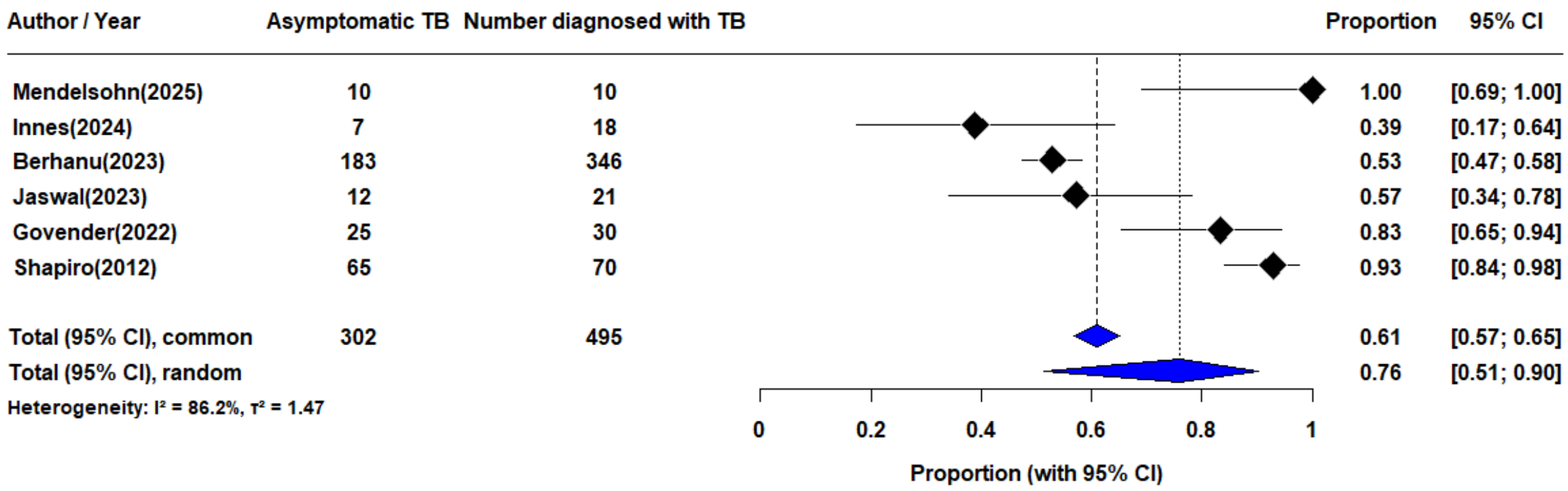

Figure S21. Pooled proportion of asymptomatic TB among studies that included household contact in the screening process

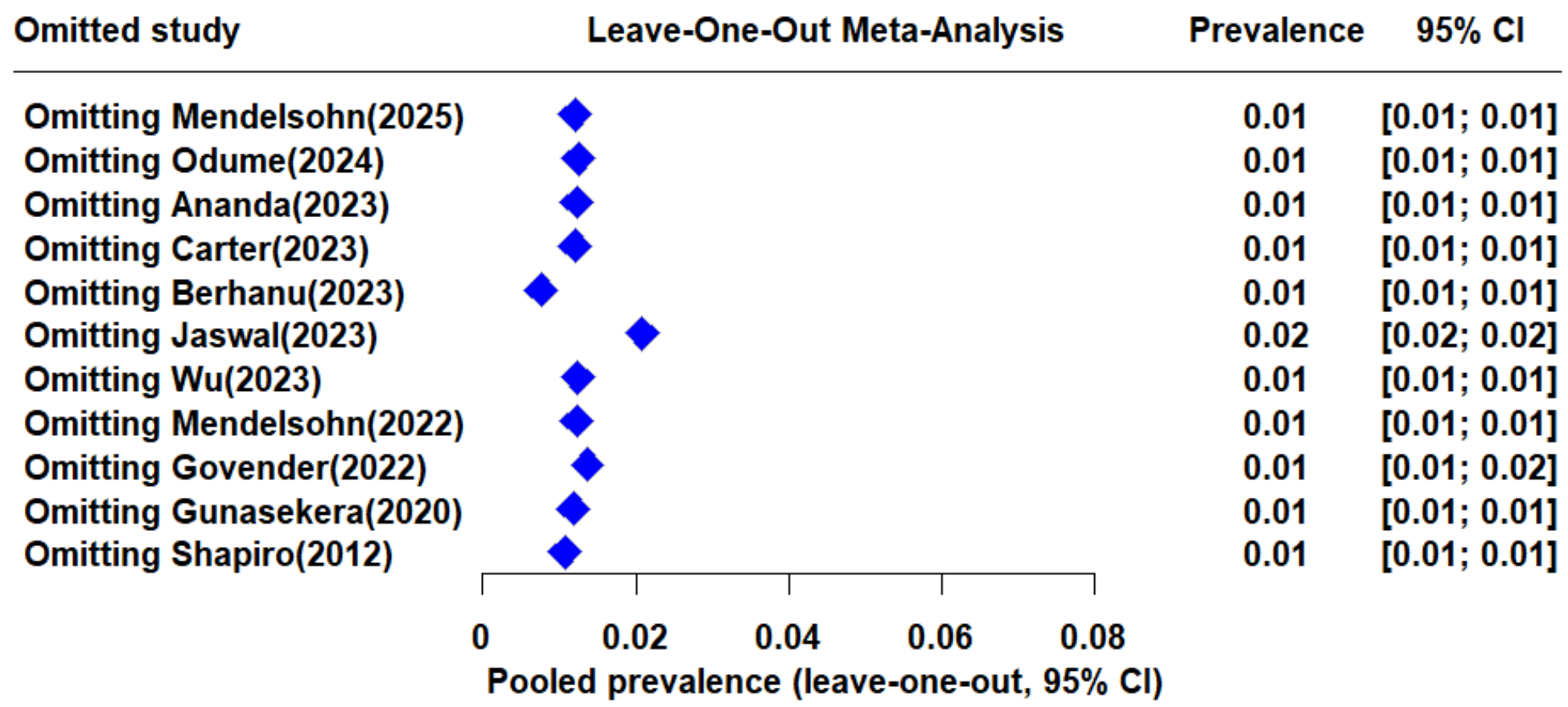

Figure S22. Leave-one-out analysis for prevalence estimation

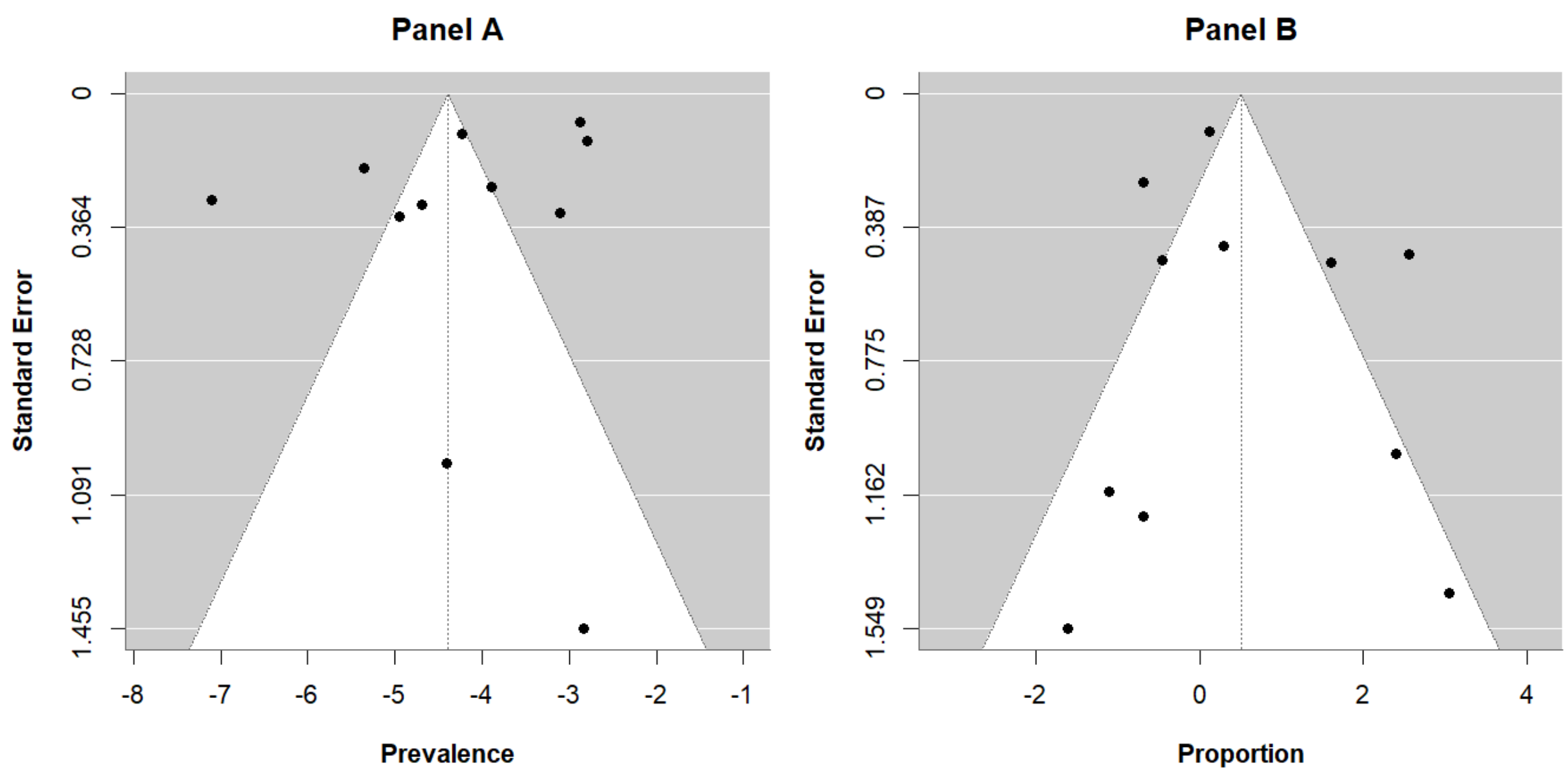

Figure S23. Funnel plots of (a) the prevalence of TB in AYAs and (b) the proportion of asymptomatic TB among AYAs with TB

Sensitivity search strategy

Following the reviewer's suggestion that the original search strategy may have been too narrow, we conducted a broader sensitivity search. This search included additional terms related to adults, active case finding, systematic screening, and community- or household-based TB screening to identify studies that may not have used the terms "adolescent," "asymptomatic," or "prevalence." The sensitivity search identified one additional eligible study, which was included in a sensitivity meta-analysis.

PUBMED

"("Tuberculosis"[MeSH Terms] OR ("tuberculosi"[All Fields] OR "Tuberculosis"[MeSH Terms] OR "Tuberculosis"[All Fields] OR "tuberculoses"[All Fields] OR "tuberculosis s"[All Fields]) OR "TB"[All Fields] OR "Mycobacterium tuberculosis"[All Fields]) AND ("Mass Screening"[MeSH Terms] OR "Contact Tracing"[MeSH Terms] OR "screen"[All Fields] OR "active case finding"[All Fields] OR "case finding"[All Fields] OR "systematic screening"[All Fields] OR "contact investigation"[All Fields] OR "household contact"[All Fields] OR "household contacts"[All Fields] OR "community-based screening"[All Fields] OR "community screening"[All Fields] OR "population-based survey"[All Fields] OR "population-based surveys"[All Fields] OR "prevalence survey"[All Fields] OR "prevalence surveys"[All Fields] OR "door-to-door"[All Fields] OR ("yield"[All Fields] OR "yielded"[All Fields] OR "yielding"[All Fields] OR "yields"[All Fields])) AND ("asymptomatic"[All Fields] OR "asymptomatically"[All Fields] OR "asymptomatics"[All Fields] OR ("subclinic"[All Fields] OR "subclinical"[All Fields] OR "subclinically"[All Fields] OR "subclinicals"[All Fields]) OR "subclinical tuberculosis"[All Fields] OR "subclinical TB"[All Fields] OR "no symptom"[All Fields] OR "no symptoms"[All Fields] OR "without symptom"[All Fields] OR "without symptoms"[All Fields] OR "absence of symptoms"[All Fields] OR "symptom-negative"[All Fields] OR "symptom-negative"[All Fields] OR "not reporting symptoms"[All Fields] OR "did not report symptoms"[Title/Abstract:~0] OR "undiagnosed"[All Fields] OR "undiagnosed tuberculosis"[All Fields] OR "undiagnosed TB"[All Fields] OR "screen-detected"[All Fields] OR "screen detect*"[All Fields] OR "bacteriologically confirmed"[All Fields] OR "culture-positive"[All Fields] OR "culture-positive"[All Fields] OR "xpert positive"[All Fields] OR "xpert positive"[All Fields] OR "smear-positive"[All Fields] OR "smear-positive"[All Fields]) AND ("Adolescent"[MeSH Terms] OR "Young Adult"[MeSH Terms] OR ("adolescences"[All Fields] OR "adolescency"[All Fields] OR "Adolescent"[MeSH Terms] OR "Adolescent"[All Fields] OR "adolescence"[All Fields] OR "adolescents"[All Fields] OR "adolescent s"[All Fields]) OR ("adolescences"[All Fields] OR "adolescency"[All Fields] OR "Adolescent"[MeSH Terms] OR "Adolescent"[All Fields] OR "adolescence"[All Fields] OR "adolescents"[All Fields] OR "adolescent s"[All Fields]) OR ("adolescences"[All Fields] OR "adolescency"[All Fields] OR "Adolescent"[MeSH Terms] OR "Adolescent"[All Fields] OR "adolescence"[All Fields] OR "adolescents"[All Fields] OR "adolescent s"[All Fields]) OR*

("Adolescent"[MeSH Terms] OR "Adolescent"[All Fields] OR "youth"[All Fields] OR "youths"[All Fields] OR "youth s"[All Fields]) OR ("Adolescent"[MeSH Terms] OR "Adolescent"[All Fields] OR "youth"[All Fields] OR "youths"[All Fields] OR "youth s"[All Fields]) OR "young people"[All Fields] OR "young person"[All Fields] OR "Young Adult"[All Fields] OR "young adults"[All Fields] OR ("Adolescent"[MeSH Terms] OR "Adolescent"[All Fields] OR "teenage"[All Fields] OR "teenager"[All Fields] OR "teenagers"[All Fields] OR "teenaged"[All Fields] OR "teenager s"[All Fields] OR "teenages"[All Fields]) OR ("Adolescent"[MeSH Terms] OR "Adolescent"[All Fields] OR "teenage"[All Fields] OR "teenager"[All Fields] OR "teenagers"[All Fields] OR "teenaged"[All Fields] OR "teenager s"[All Fields] OR "teenages"[All Fields]) OR ("Adolescent"[MeSH Terms] OR "Adolescent"[All Fields] OR "teen"[All Fields]) OR ("Adolescent"[MeSH Terms] OR "Adolescent"[All Fields] OR "teens"[All Fields] OR "teen s"[All Fields]) OR ("student s"[All Fields] OR "students"[MeSH Terms] OR "students"[All Fields] OR "student"[All Fields] OR "students s"[All Fields]) OR ("student s"[All Fields] OR "students"[MeSH Terms] OR "students"[All Fields] OR "student"[All Fields] OR "students s"[All Fields]) OR "10-19"[All Fields] OR "10-24"[All Fields] OR "10-25"[All Fields] OR "15-24"[All Fields] OR "15-25"[All Fields])"

Sensitivity meta-analysis with one new included study

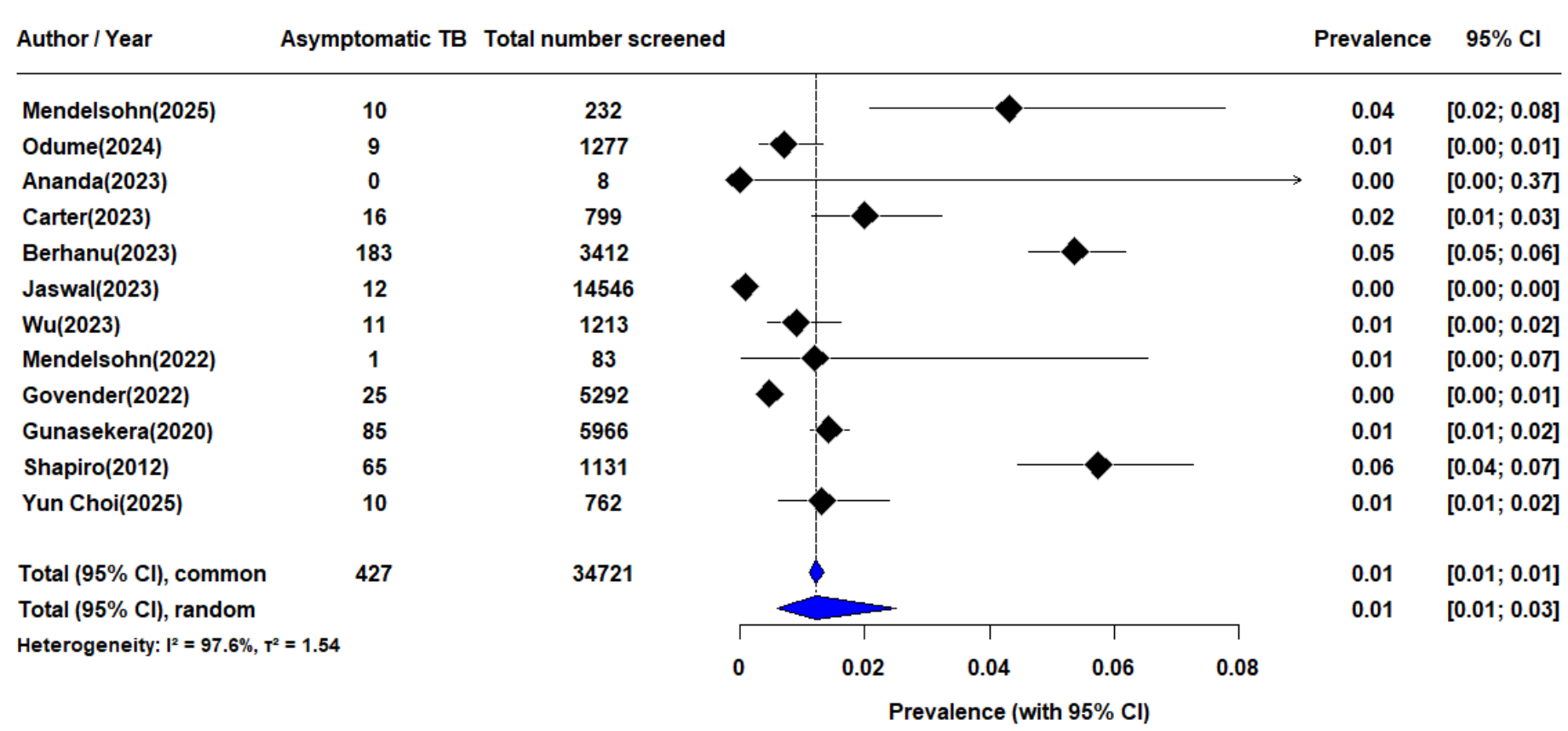

Author / Year	Asymptomatic TB	Number diagnosed with TB	Proportion	95% CI
Mendelsohn(2025)	10	10	1.00	[0.69; 1.00]
Innes(2024)	7	18	0.39	[0.17; 0.64]
Ananda(2023)	0	2	0.00	[0.00; 0.84]
Wang(2023)	1	3	0.33	[0.01; 0.91]
Berhanu(2023)	183	346	0.53	[0.47; 0.58]
Jaswal(2023)	12	21	0.57	[0.34; 0.78]
Wu(2023)	11	12	0.92	[0.62; 1.00]
Govender(2022)	25	30	0.83	[0.65; 0.94]
Dorjee(2021)	23	69	0.33	[0.22; 0.46]
Nair(2016)	1	3	0.33	[0.01; 0.91]
Shapiro(2012)	65	70	0.93	[0.84; 0.98]
Da Costa(2011)	1	4	0.25	[0.01; 0.81]
Yun Choi(2025)	10	14	0.71	[0.42; 0.92]
Total (95% CI), common	349	602	0.58	[0.54; 0.62]
Total (95% CI), random			0.63	[0.44; 0.80]

Heterogeneity: $I^2 = 80.1\%$, $\tau^2 = 1.84$

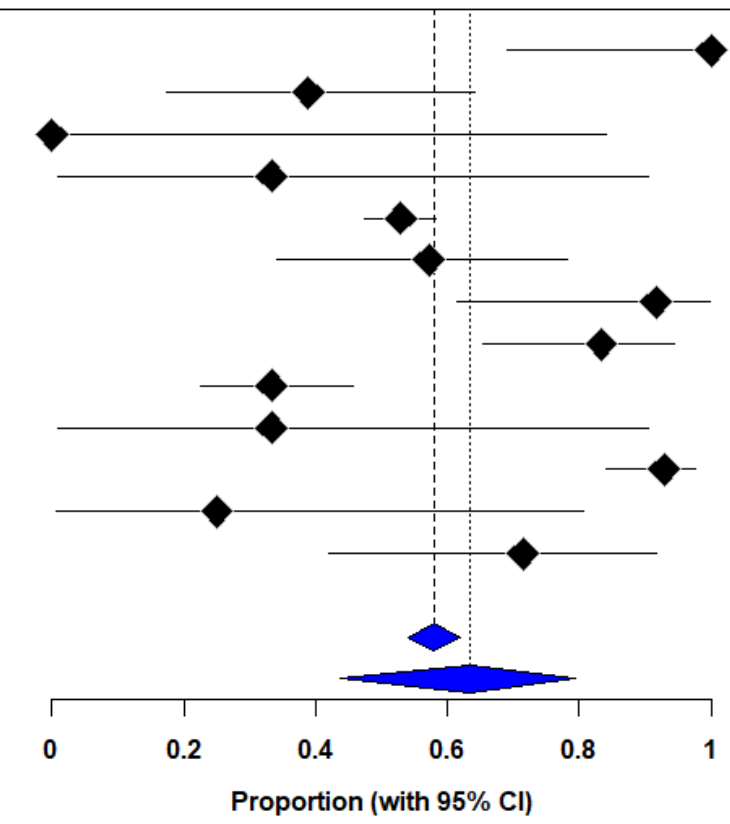

Supplement: Supplementary file 1 [file 12879_2026_14156_MOESM1_ESM.pdf]
